# Corrigendum: Quantitative real-time imaging of glutathione

**DOI:** 10.1038/ncomms16163

**Published:** 2017-10-03

**Authors:** Xiqian Jiang, Jianwei Chen, Aleksandar Bajić, Chengwei Zhang, Xianzhou Song, Shaina L. Carroll, Zhao-Lin Cai, Meiling Tang, Mingshan Xue, Ninghui Cheng, Christian P. Schaaf, Feng Li, Kevin R. MacKenzie, Allan Chris M. Ferreon, Fan Xia, Meng C. Wang, Mirjana Maletić-Savatić, Jin Wang

Nature Communications 8: Article number: 16087 ; DOI: 10.1038/ncomms16087 (2017); Published 06 13 2017; Updated 10 03 2017.

Previous work by Cho and Choi describing the development of a cyanoacrylamide-based fluorescence sensor for reversible detection of thiols in homogenous solutions was inadvertently omitted from the reference list of this Article. This work should have been cited in the first paragraph of the discussion, following the rationale behind the development of the Michael acceptor, as follows: ‘A fluorescent sensor based on the cyanoacrylamide Michael acceptor has previously been shown to reversibly react with thiols in homogenous solutions but without any cellular applications, possibly due to the low quantum yield and poor aqueous solubility (Cho *et al.*, 2012)’.

Cho, A. Y. & Choi, K. A coumarin-based fluorescence sensor for the reversible detection of thiols. *Chem. Lett*. **41**, 1611–1612 (2012).

