# Supplementary material for: Quantitative real-time imaging of glutathione
Source: Nat Commun. 2017 Jul 13;8:16087. doi: 10.1038/ncomms16087 (PMC5511354; doi:10.1038/ncomms16087)
Supplement: Supplementary Information [file ncomms16087-s1.pdf]

Type of file: PDF
Size of file: 0 KB
Title of file for HTML: Supplementary Information
Description: Supplementary Figures, Supplementary Tables and Supplementary Notes

Type of file: PDF
Size of file: 0 KB
Title of file for HTML: Peer Review File
Description:

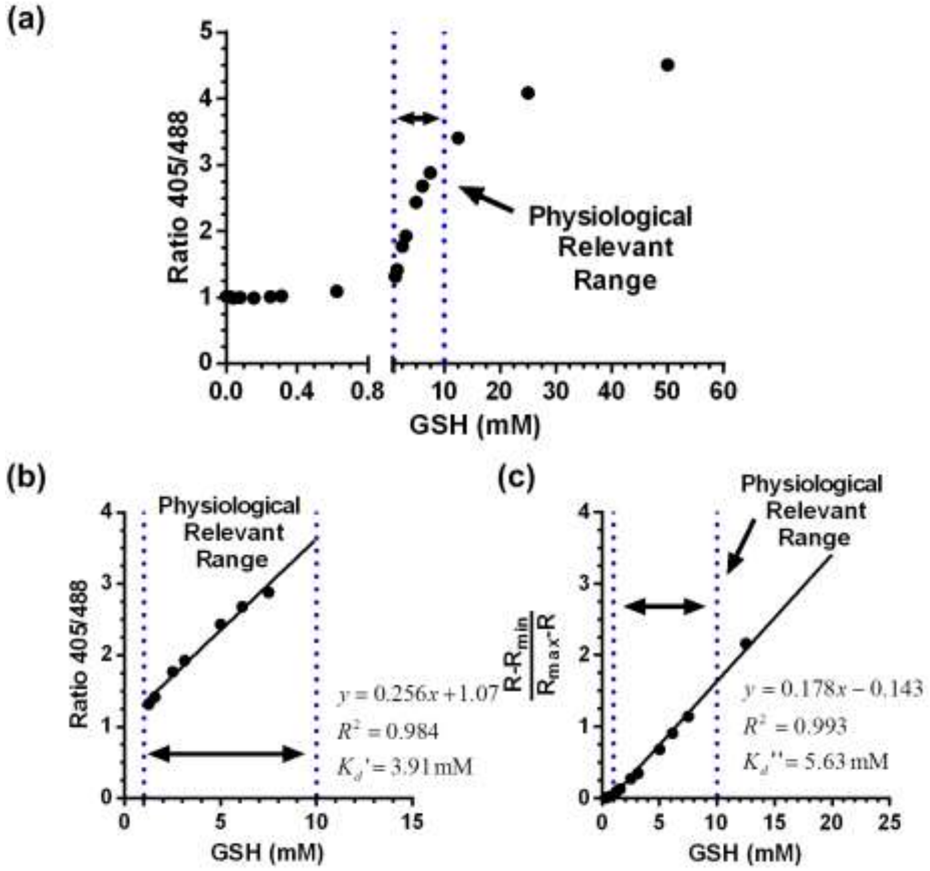

Supplementary Figure 1. Equilibrium Constant of Reaction between RT and GSH. Ratios as a function of GSH concentrations were measured using a plate reader: **(a)** full range, **(b)** linear range based on the fluorescence signal ratios (F_{405}/F_{488}), and **(c)** linear range based on the corrected ratio: (R-R_{min})/(R_{max}-R), in which R equals to F_{405}/F_{488}. Using a corrected ratio can minimize interference from spectral overlap from two species, and therefore extend the linear range and fitting quality. However, in some of our imaging studies, fluorescence signal ratios (F_{405}/F_{488}) were used instead of (R-R_{min})/(R_{max}-R). This is because that calibration curves were close to linear in the working range under the specific microscope settings. Because the reversible sensing reaction has an equilibrium constant of 3.7 mM, the RT probe is not responsive to thiol concentrations below 0.5 mM, such as cysteine at its physiological relevant concentrations. Therefore, RT has a suitable equilibrium constant for monitoring intracellular GSH levels. It should be noted that plotting R and (R-R_{min})/(R_{max}-R) against GSH concentrations will yield K_d' and K_d'', respectively, which have different physical meanings. For explanation on K_d' and K_d'', please refer to Supplementary Discussion. Each data point represents the mean value of three replicates. Error bars represent s.e.m. and are too small to show on the graph.

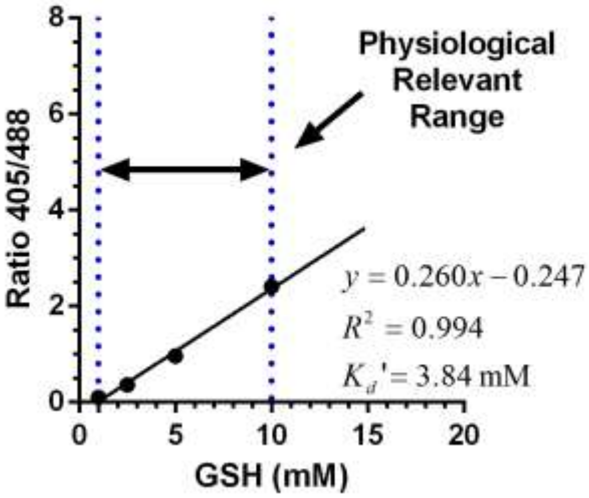

Supplementary Figure 2. Representative Calibration Curve under Scanning Confocal Microscope. Ratio F_{405}/F_{488} as a function of GSH concentration measured using a confocal microscope. This is a representative calibration curve specifically generated for quantifying neuron imaging data in Fig 2b. The physiological relevant range is shown. GSH solutions (0-50 mM in PBS (10 mM, pH 7.4)) were prepared and mixed with a RT solution (10 μM as the final concentration). The above solutions were further mixed with a suspension containing 5 μm polystyrene beads. Eight-well confocal imaging chambers were used to hold the solutions for confocal microscopy. The same microscope settings were adopted from the corresponding cell imaging experiments. Due to the hydrophilicity of the probe molecule, beads merely serve as focal points for the confocal imaging. Quantitation is based on the fluorescence intensities of the solution from both channels. For explanation on K_d', please refer to Supplementary Discussion. Each data point represents the mean value of 10 points analyzed from three independent images acquired during experiments; error bars represent s.e.m. and are too small to show on the graph.

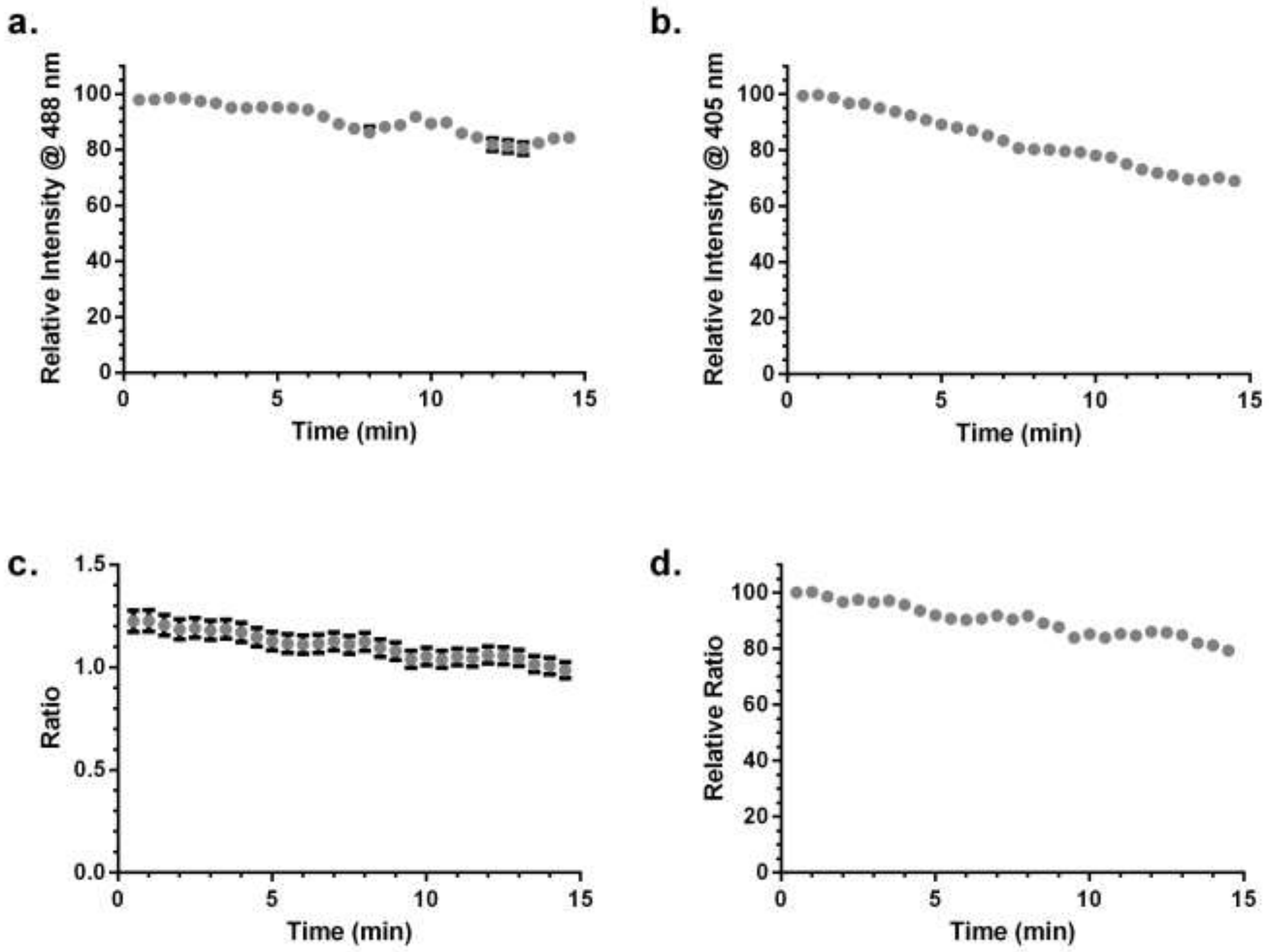

Supplementary Figure 3. Photostability of RT. HeLa cells were stained with RT-AM (1 μM) and then fixed with 4% of paraformaldehyde to prevent potential probe leaking through active clearance processes inside cells. Cells were then imaged with confocal microscopy and the corresponding ratio between the 405 nm and 488 nm channels was calculated as stated above. Each data point represents the mean value of 27 cells analyzed from three independent time-lapsed imaging experiments acquired during one representative experiment. Error bars represent s.e.m., some of which are too small to show on the graph. **(a)** Relative fluorescence intensities of the 488 nm channel. **(b)** Relative fluorescence intensities of the 405 nm channel. **(c)** Average ratios. **(d)** Relative ratios normalized by the ratio at time zero as 100%.

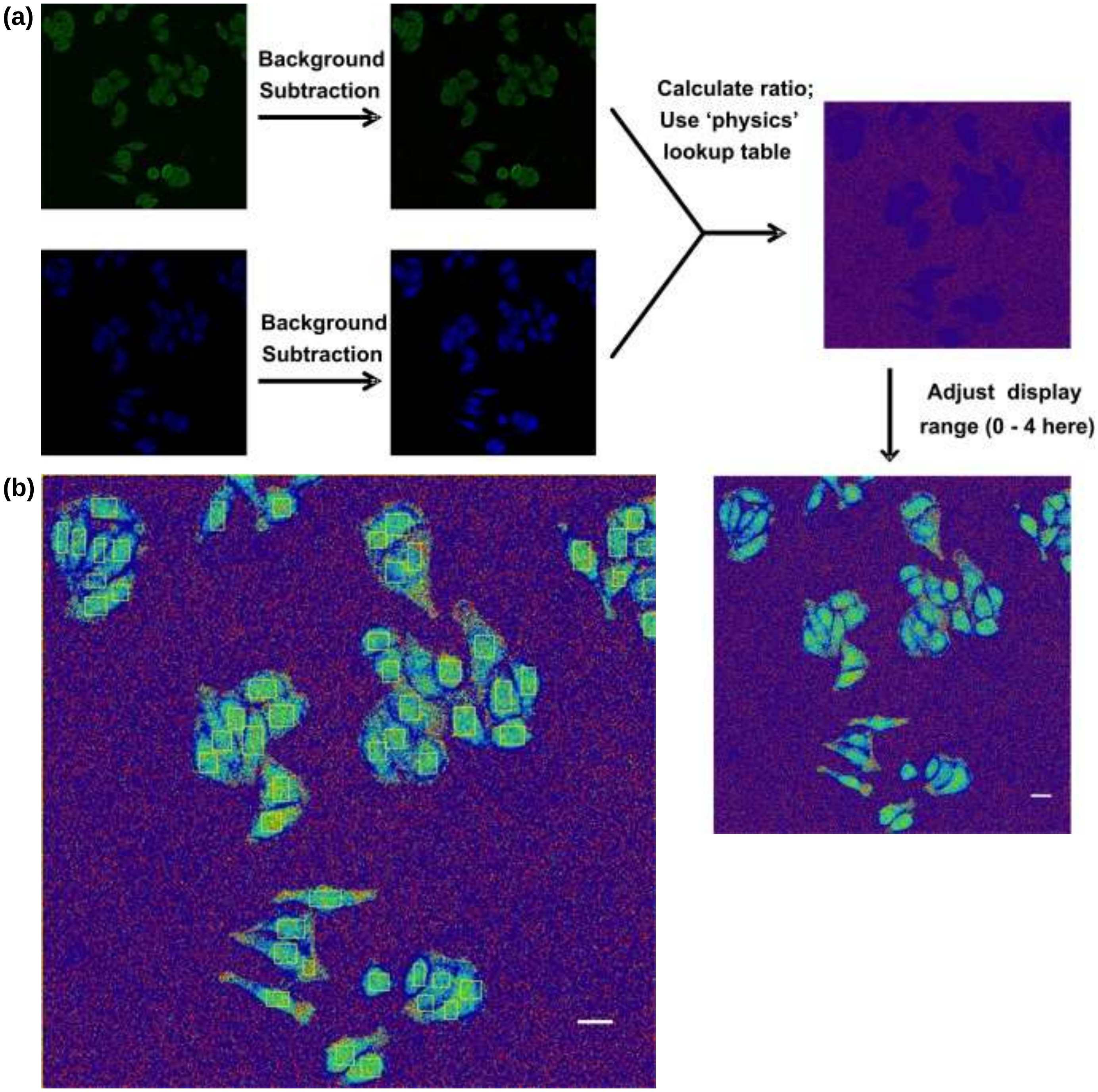

Supplementary Figure 4. Image processing for GSH ratiometric images. (a) Imaging processing procedure: ratio map generation. Images in two channels (488 and 405) were acquired at a time interval between 10-60 s. All raw images were processed using Fiji-ImageJ program. Background subtraction was performed using a rolling ball algorithm in ImageJ (refer to: http://imagej.net/Rolling_Ball_Background_Subtraction for details) with a threshold of 100 to avoid artifacts caused by uneven illumination during the confocal imaging process. Ratiometric images were generated by dividing the 405 channel image by the 488 channel image at each corresponding pixel. We used the ImageJ lookup table 'Physics' for creating the pseudo color ratiometric map. For clarity and comparison, the display range was adjusted according to the imaging settings. The example shown here adopted a display range of 0-4. For clarity, background was masked in black in Fig.2a. Scale bar 20 µm. **(b)** Imaging processing procedure: ROI assignment and ratio measurement. Data point collection is performed by randomly choosing areas in the cytosol. Typically, one to three non-overlapping areas are chosen for each cell. An example showing the selection of region of interest is presented. Scale bar: 20 µm. All the measurement results were summarized in Supplementary Table 2.

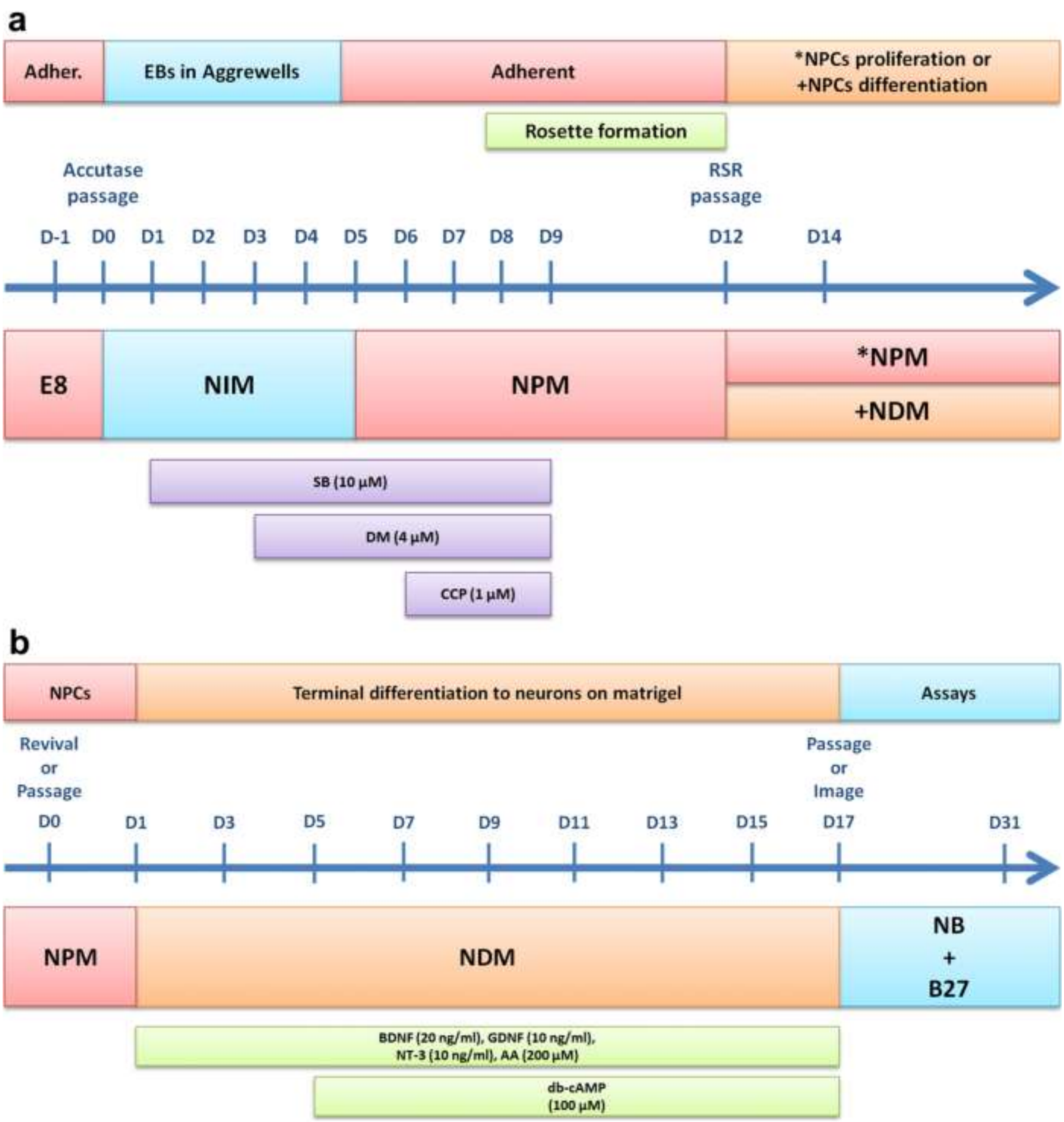

Supplementary Figure 5. **Protocols for NPC and Neurons Differentiation and Cell Culture.** Two-step protocol for differentiation of pluripotent stem cells into neurons. **(a)** Outline of utilized dual SMAD inhibition based induction of differentiation of ESCs into neural progenitors. **(b)** Terminal differentiation of NPCs into neurons.

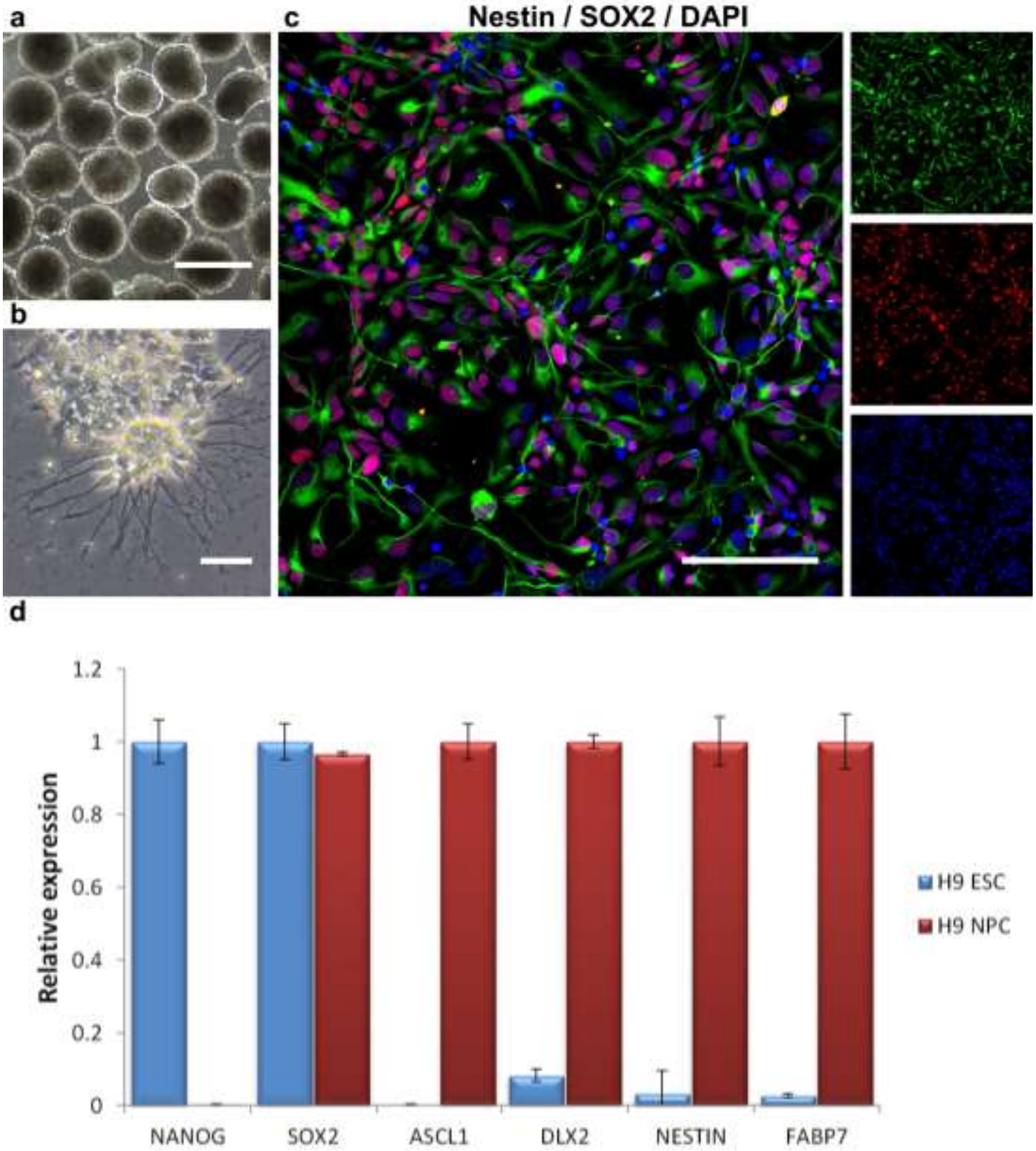

Supplementary Figure 6. Differentiation and Characterization of Neural Progenitors. (a) After initial five days of induction we collected and strained aggregates uniform in size. Scale bar 400 µm. **(b)** Example of a cell cluster enriched with NPC we harvested and plated on matrigel at day 12 of the induction protocol. Scale bar 20 µm. **(c)** Nature of the progenitors acquired by our method was confirmed by performing immunostainings for specific markers (such as NESTIN (green) and SOX2 (red)). Scale bar in (c) 100 µm. **(d)** Real-time PCR data shows a high fold change in specific NPCs markers such as ASCL1, DLX2, NES and FABP7. Each data point represents the mean value of two replicates. Error bars represent s.d. As presented, our NPCs were highly positive for all tested proteins and expression markers.

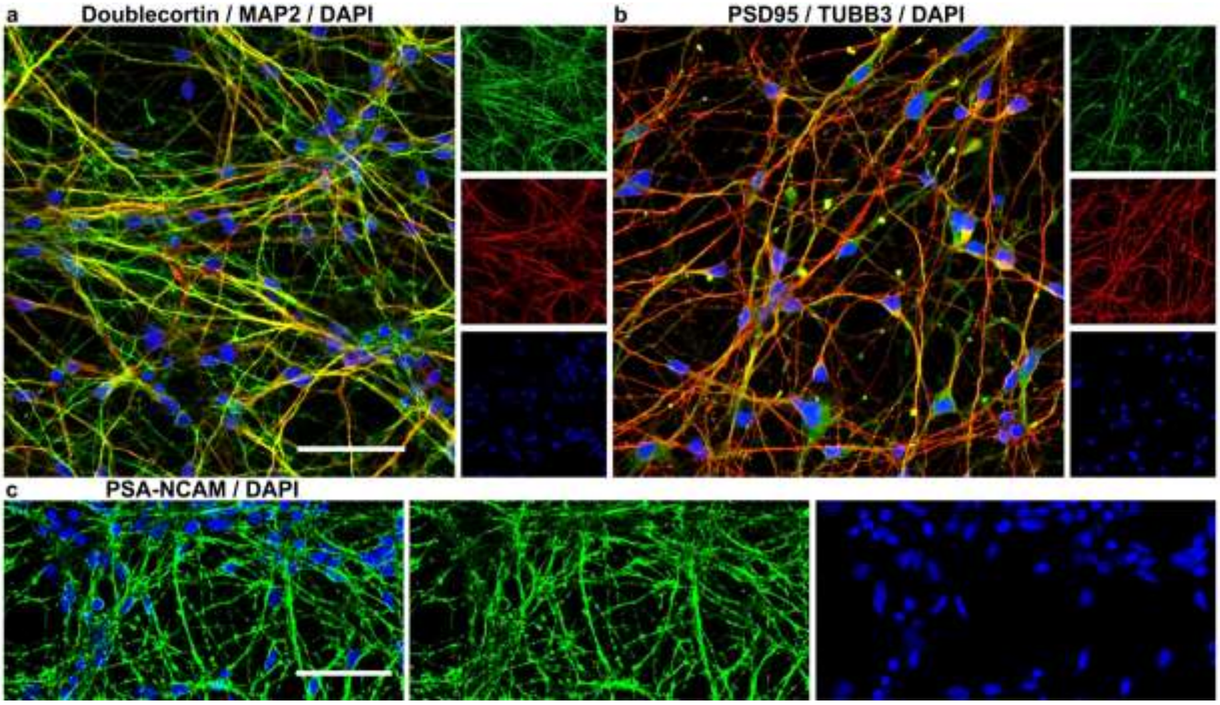

Supplementary Figure 7. Characterization of Derived Neurons by Immunocytochemistry. To confirm that terminally differentiated cells are neurons, approximately ten days after original cultures were passaged, we immunolabeled cells against **(a)** doublecortin (green) and MAP2 (red), **(b)** PSD95 (green) and tubulin beta-3 (red) and **(c)** PSA-NCAM (green). Nuclear content counterstained with DAPI. Scale bars: 50 µm.

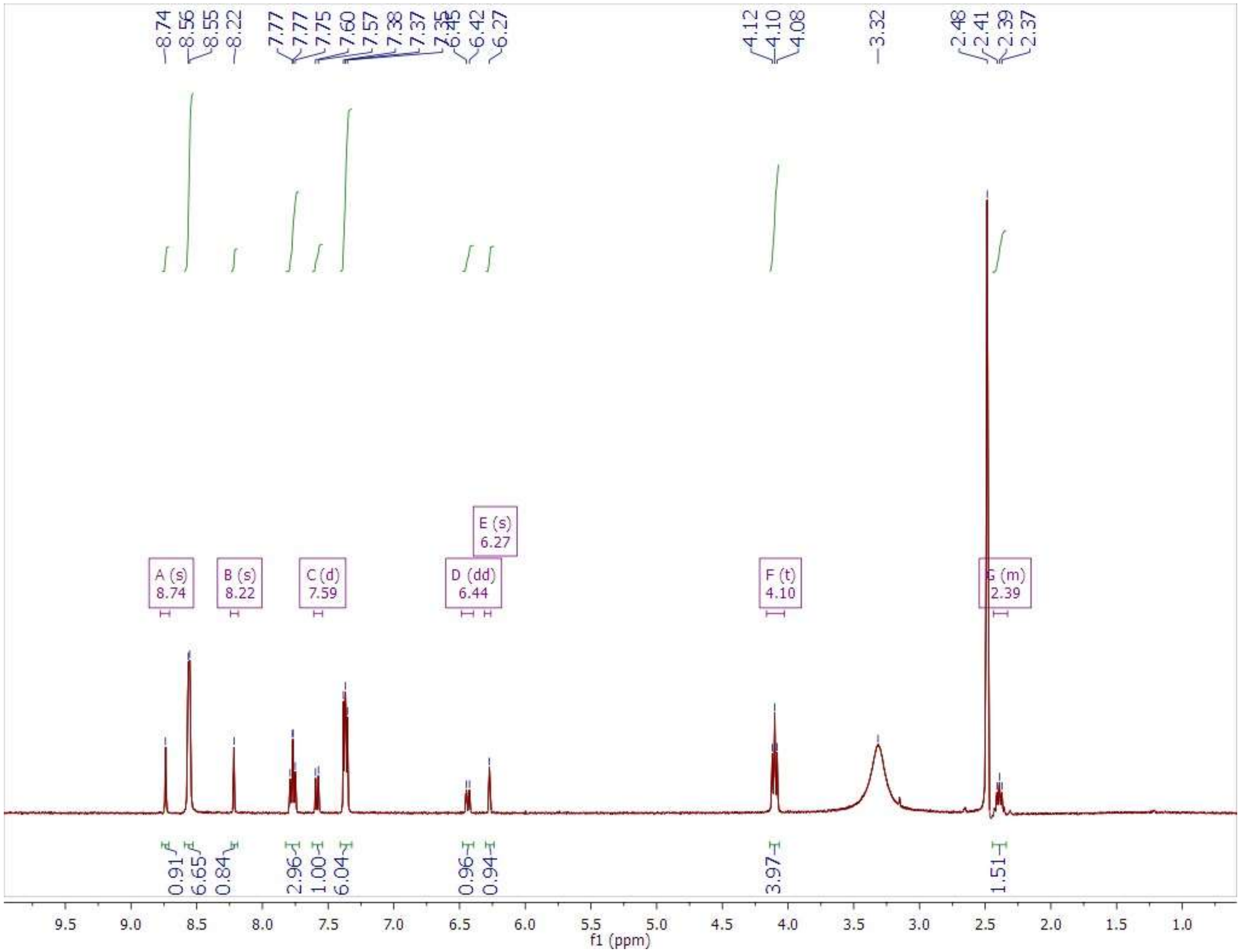

Supplementary Figure 8. ^{1}H-NMR Spectrum of Compound 2. Solvent signals from pyridine were present at δ 8.55, 7.77 and 7.37.

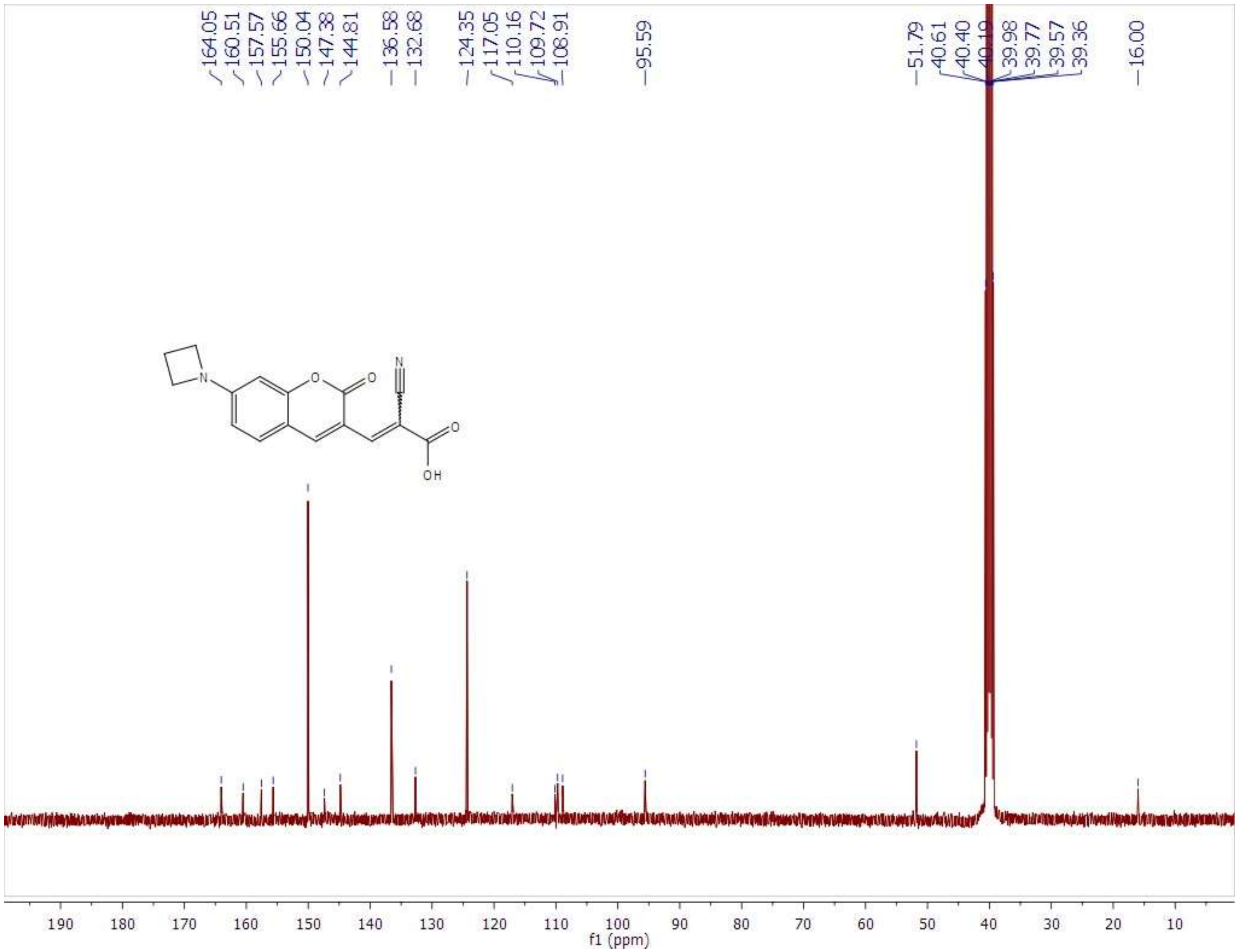

Supplementary Figure 9. ^{13}C-NMR Spectrum of Compound 2. Solvent signals from pyridine were present at δ 150.04, 136.58 and 124.35.

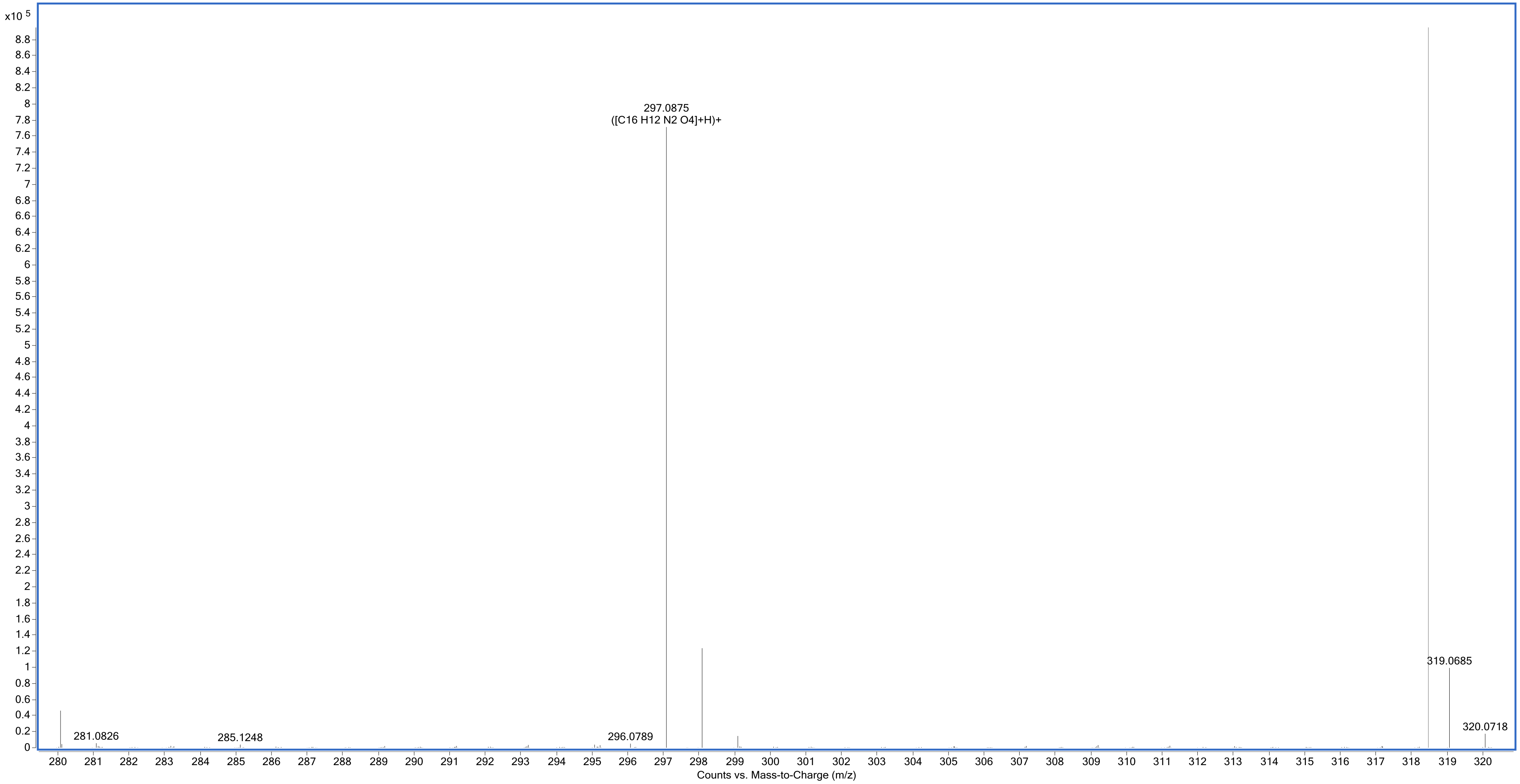

Supplementary Figure 10. HRMS Spectrum of Compound 2.

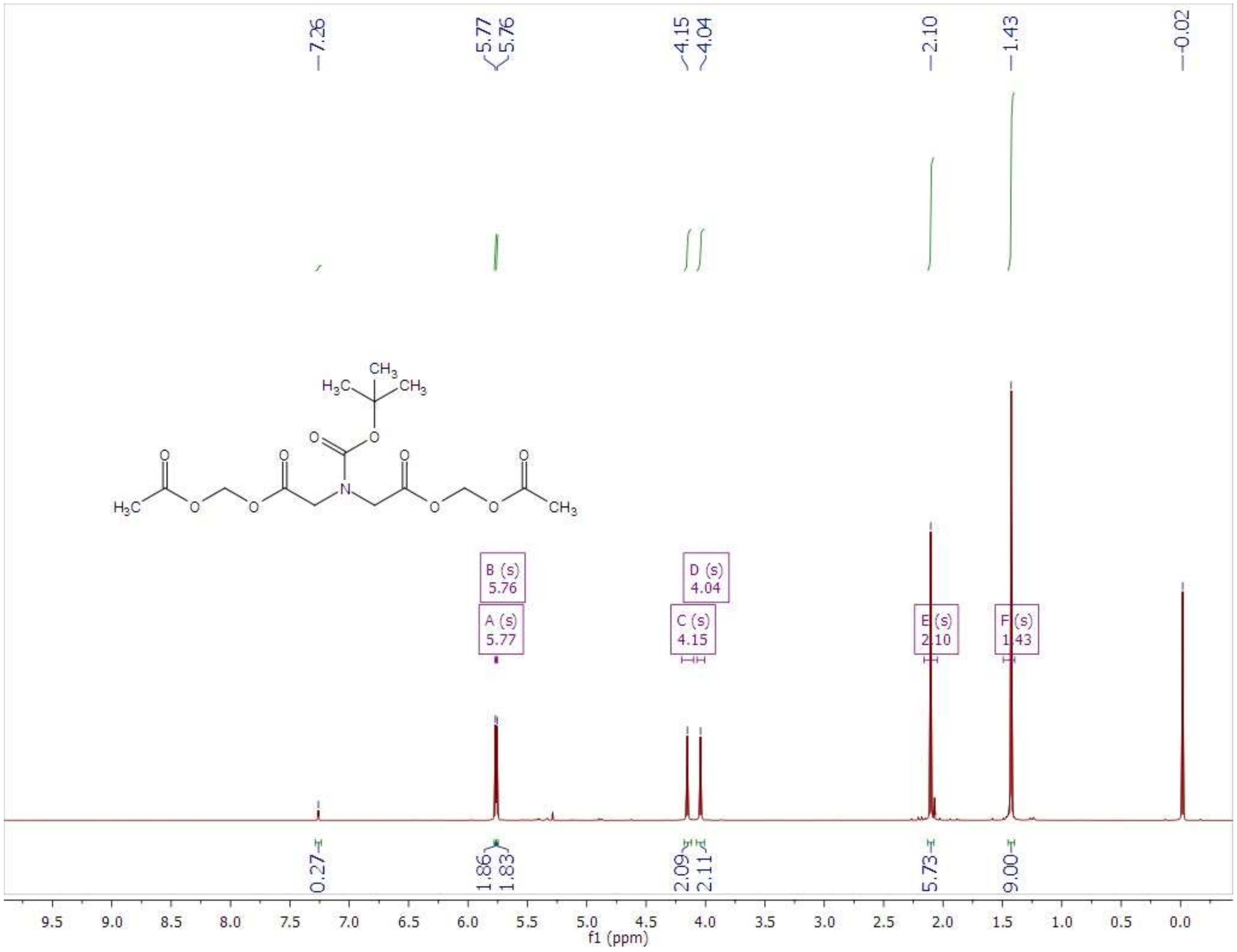

Supplementary Figure 11. ^{1}H-NMR Spectrum of Compound 4

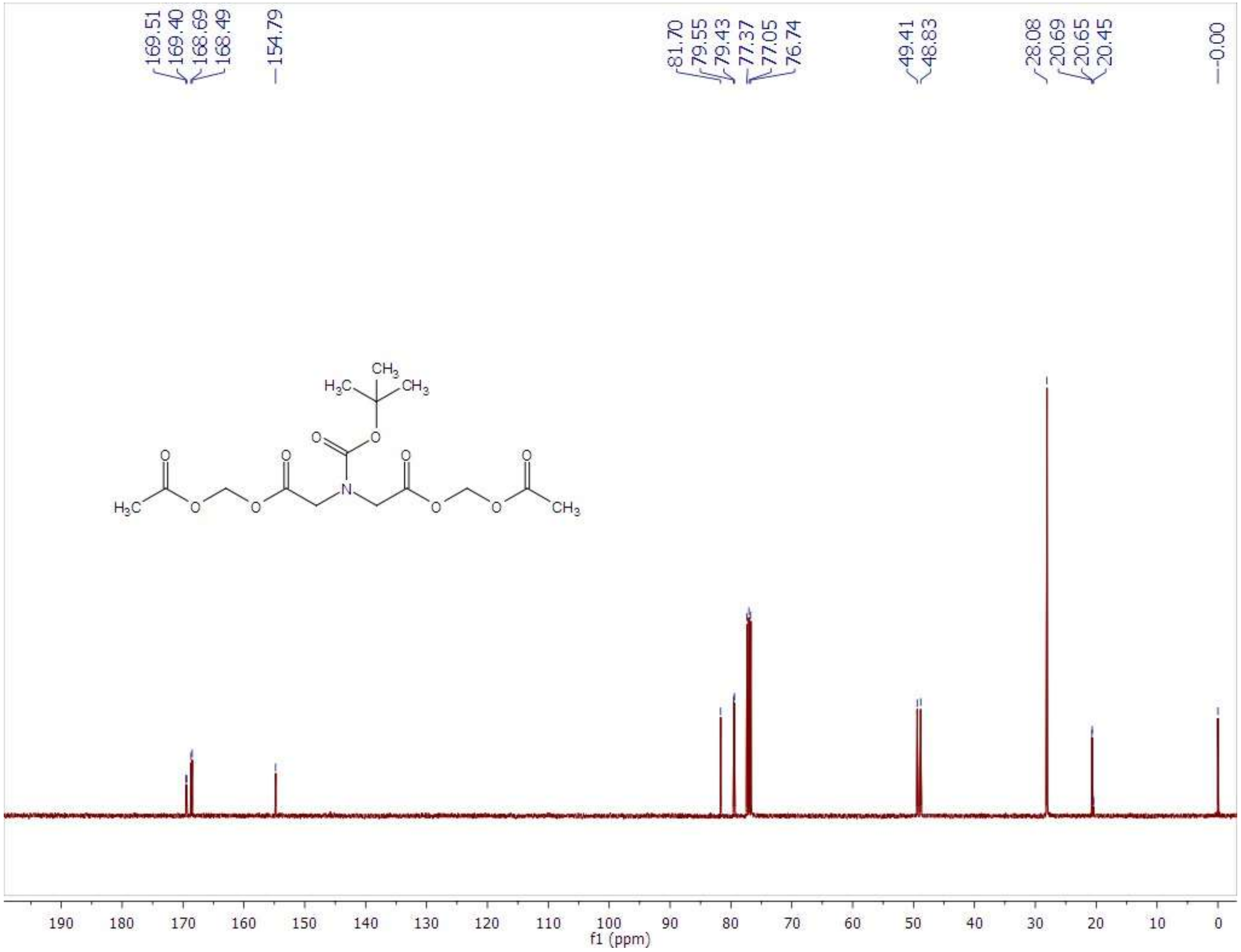

Supplementary Figure 12. ^{13}C-NMR Spectrum of Compound 4

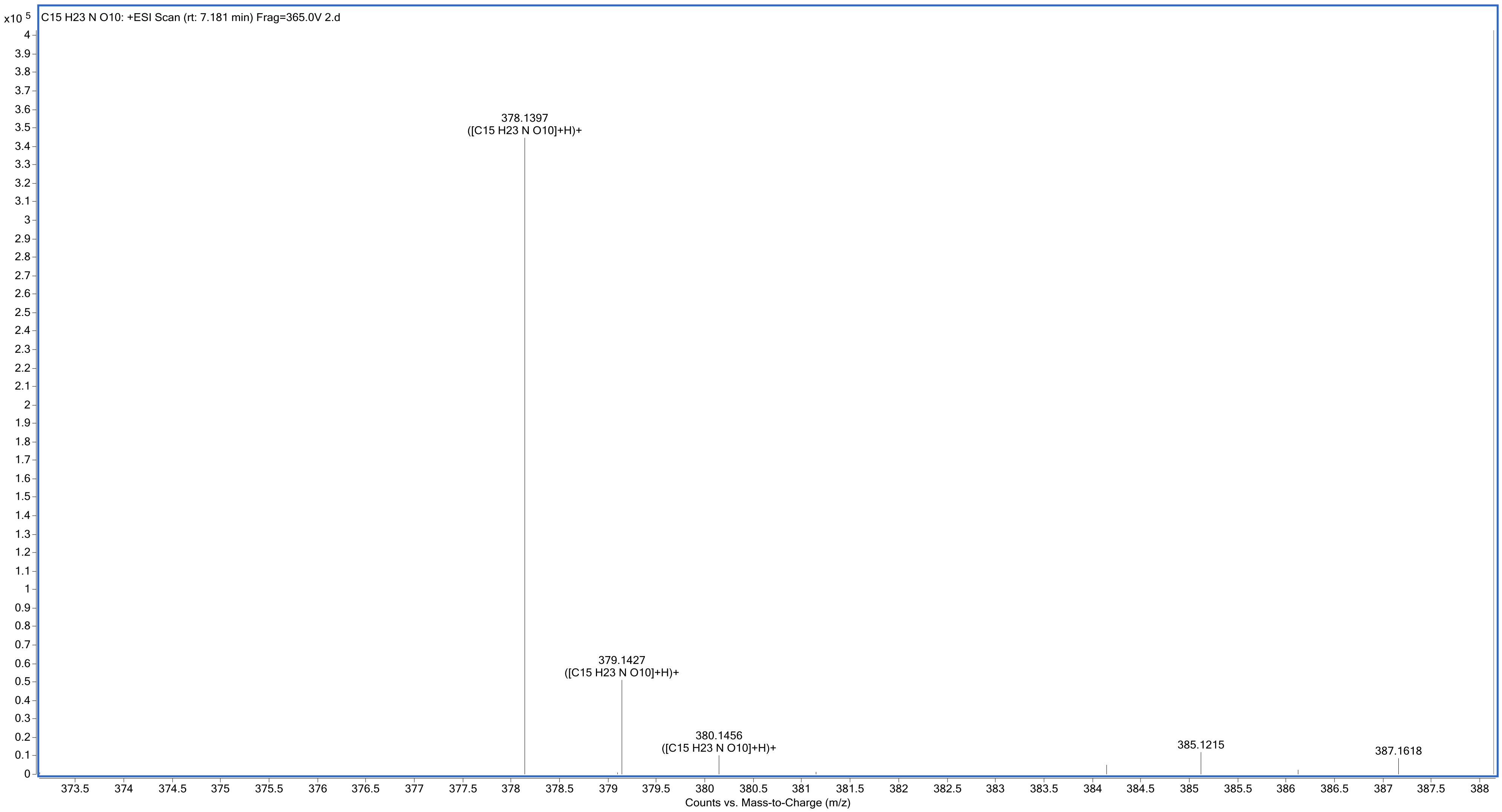

Supplementary Figure 13. HRMS Spectrum of Compound 4

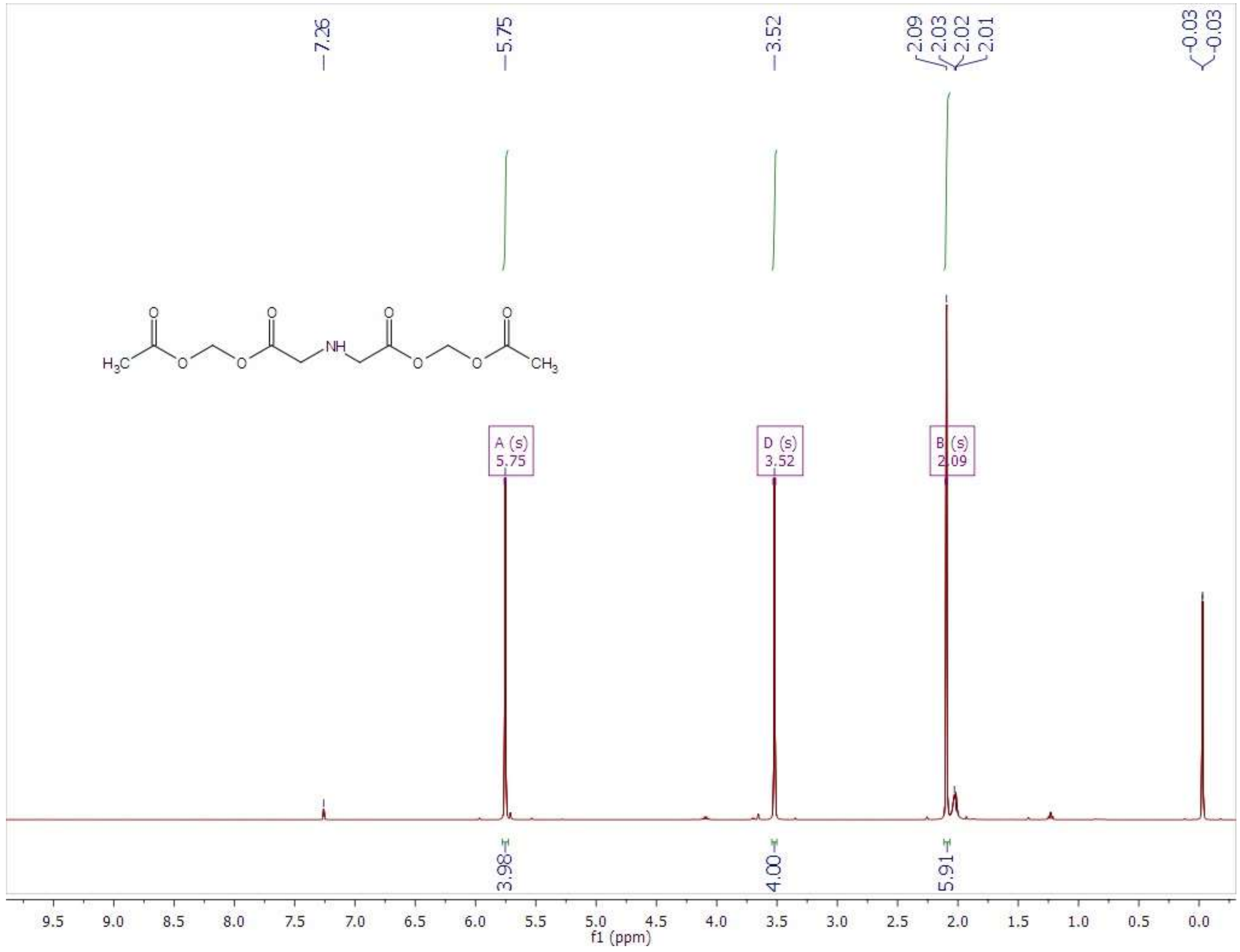

Supplementary Figure 14. ^{1}H-NMR Spectrum of Compound 5

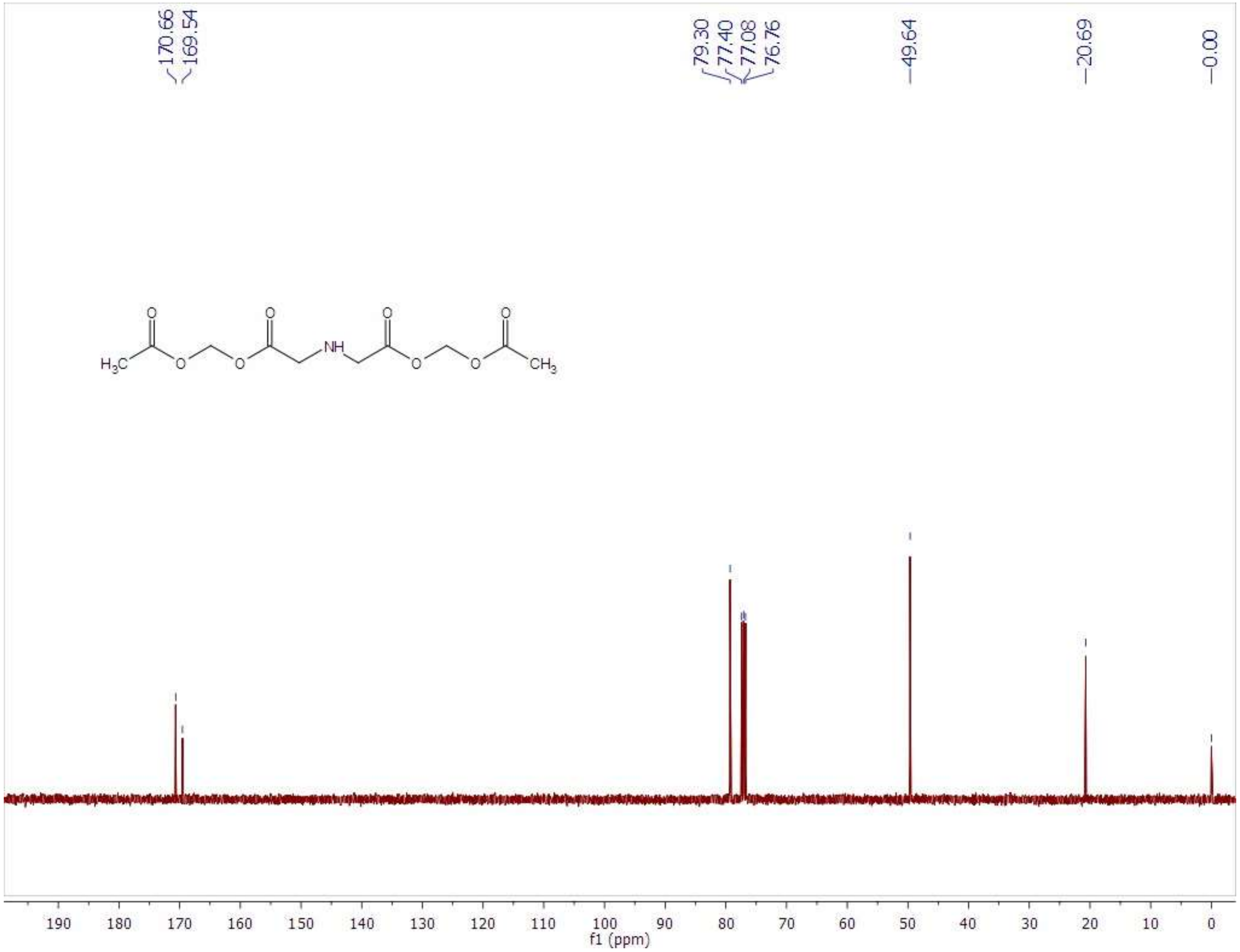

Supplementary Figure 15. ^{13}C-NMR Spectrum of Compound 5

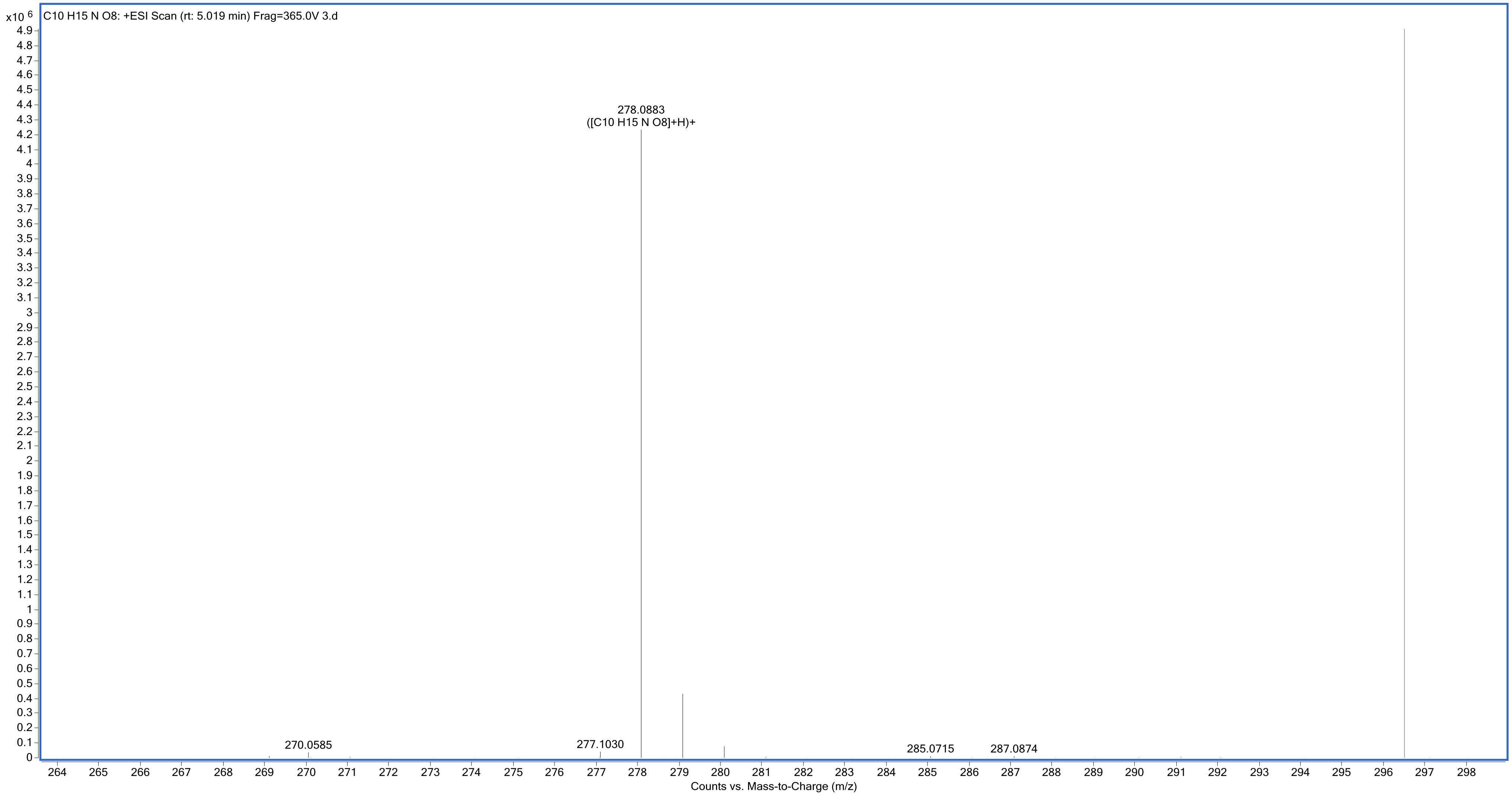

Supplementary Figure 16. HRMS Spectrum of Compound 5

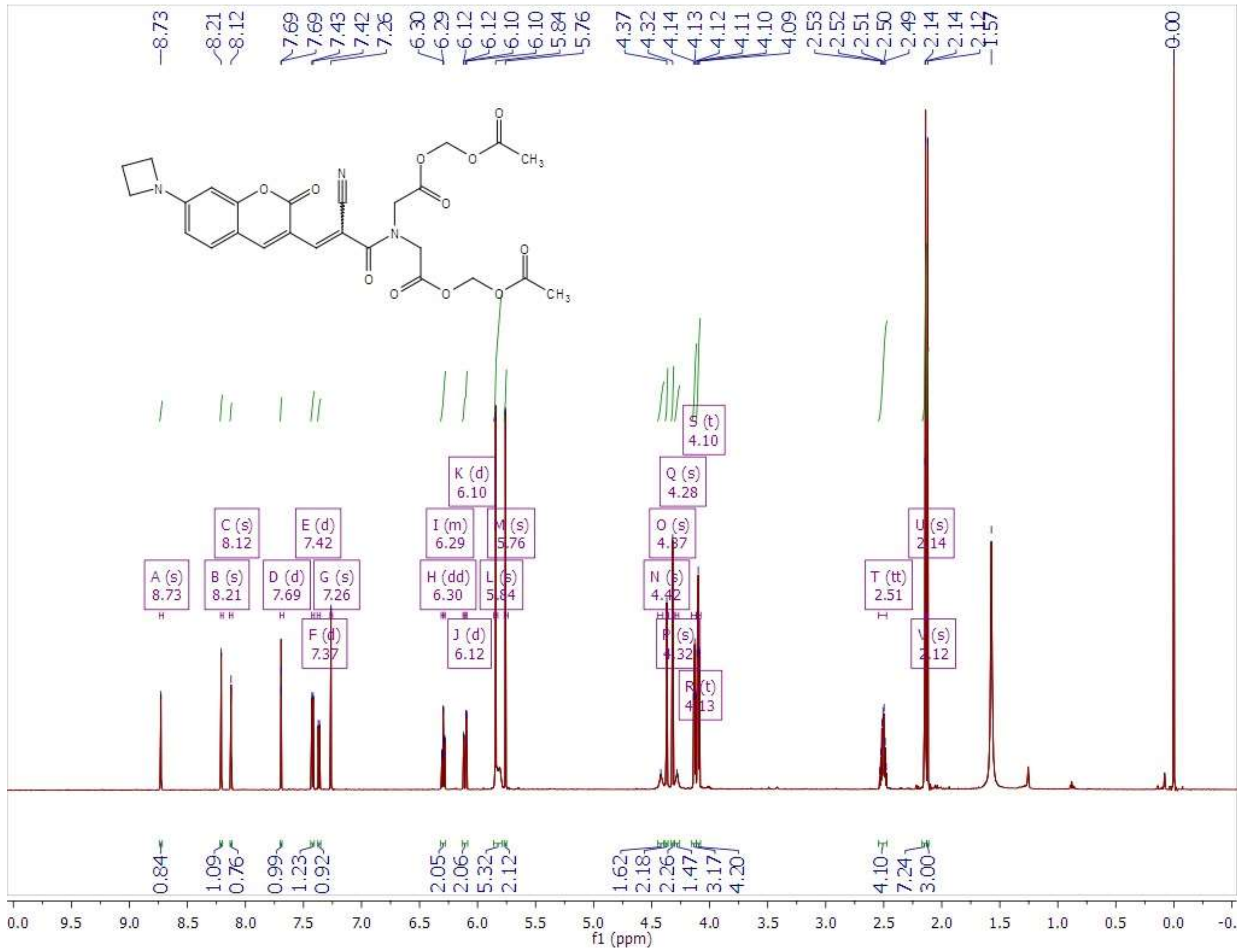

Supplementary Figure 17. ^{1}H-NMR Spectrum of RT-AM

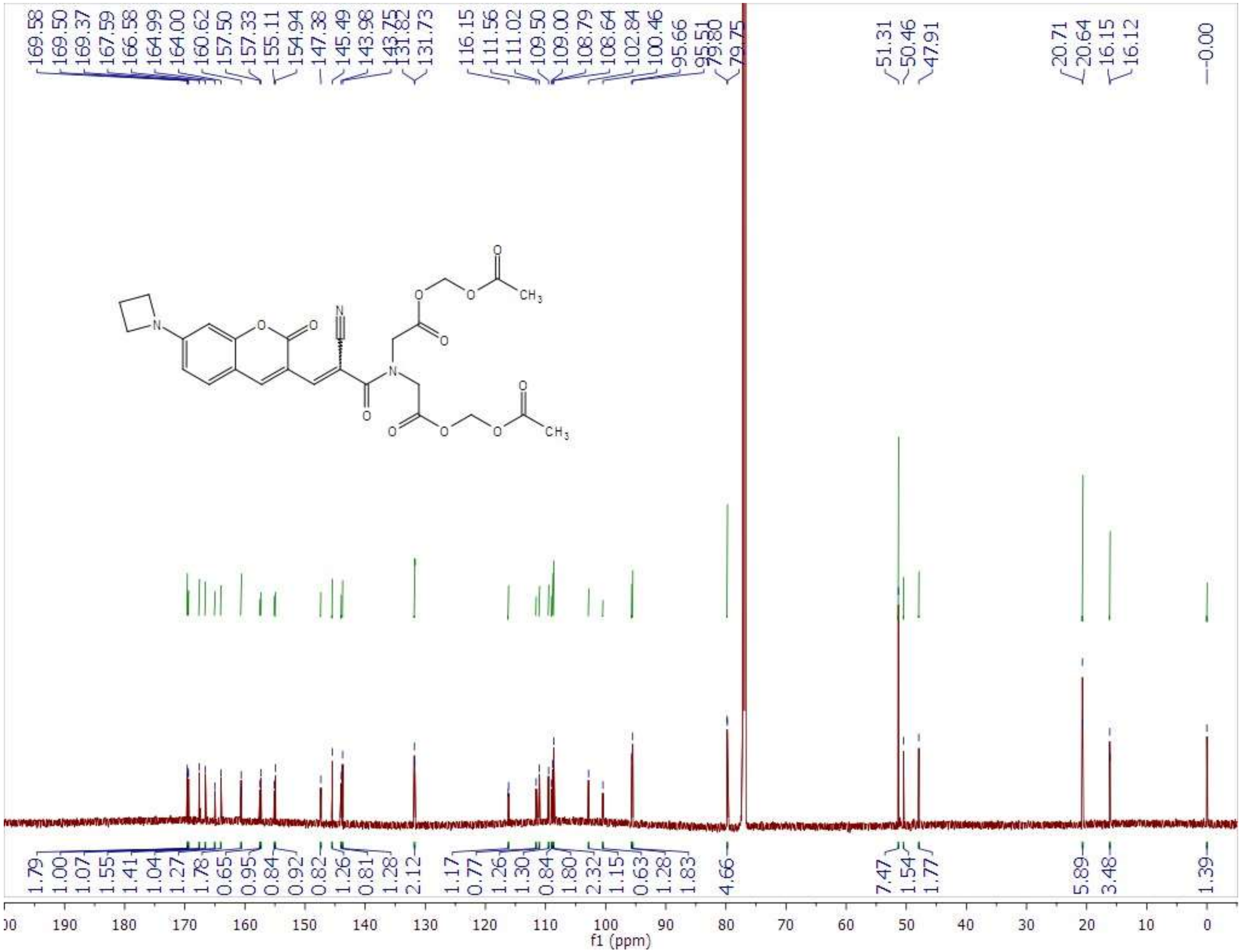

Supplementary Figure 18. ^{13}**C-NMR Spectrum of RT-AM**

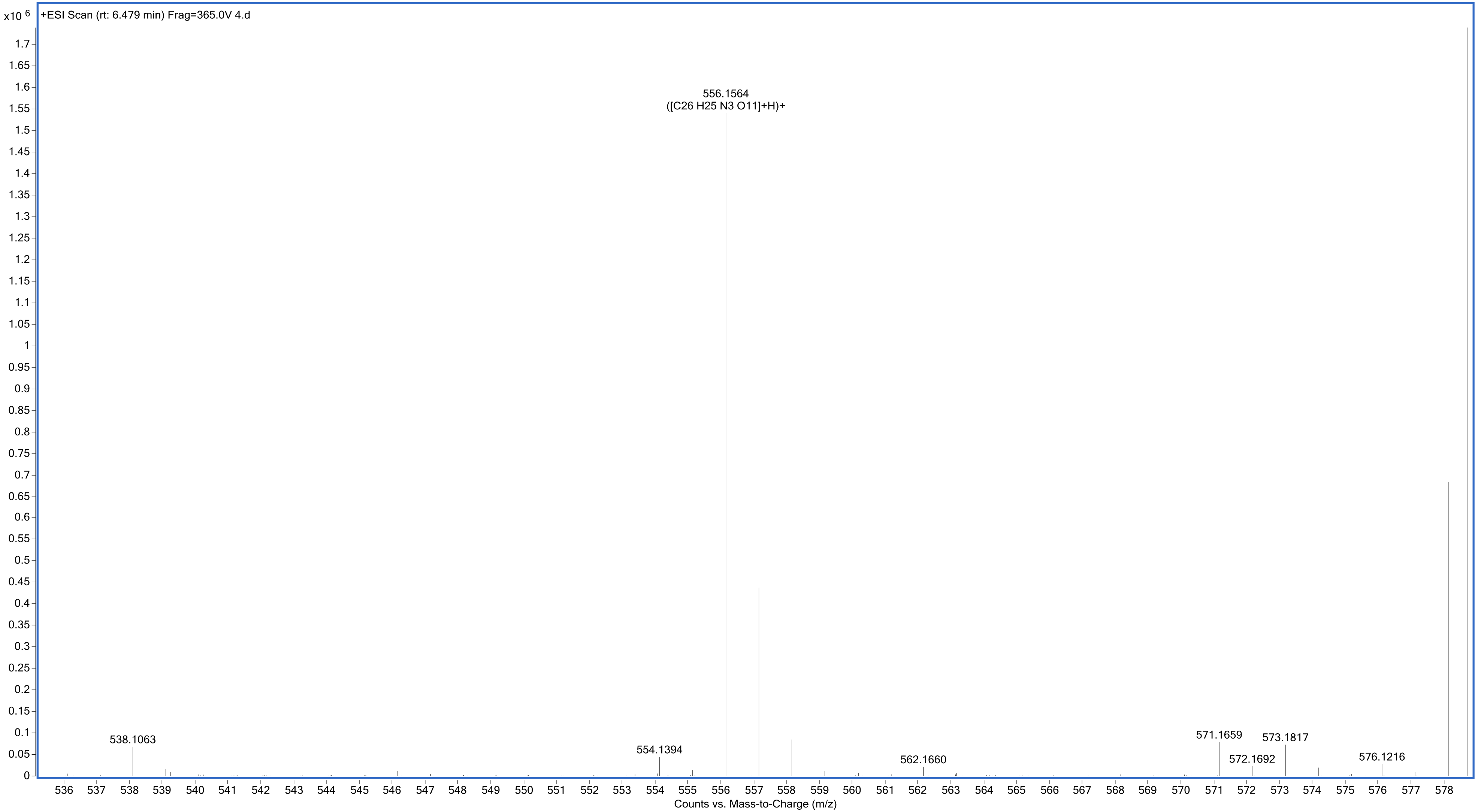

Supplementary Figure 19. HRMS Spectrum of RT-AM

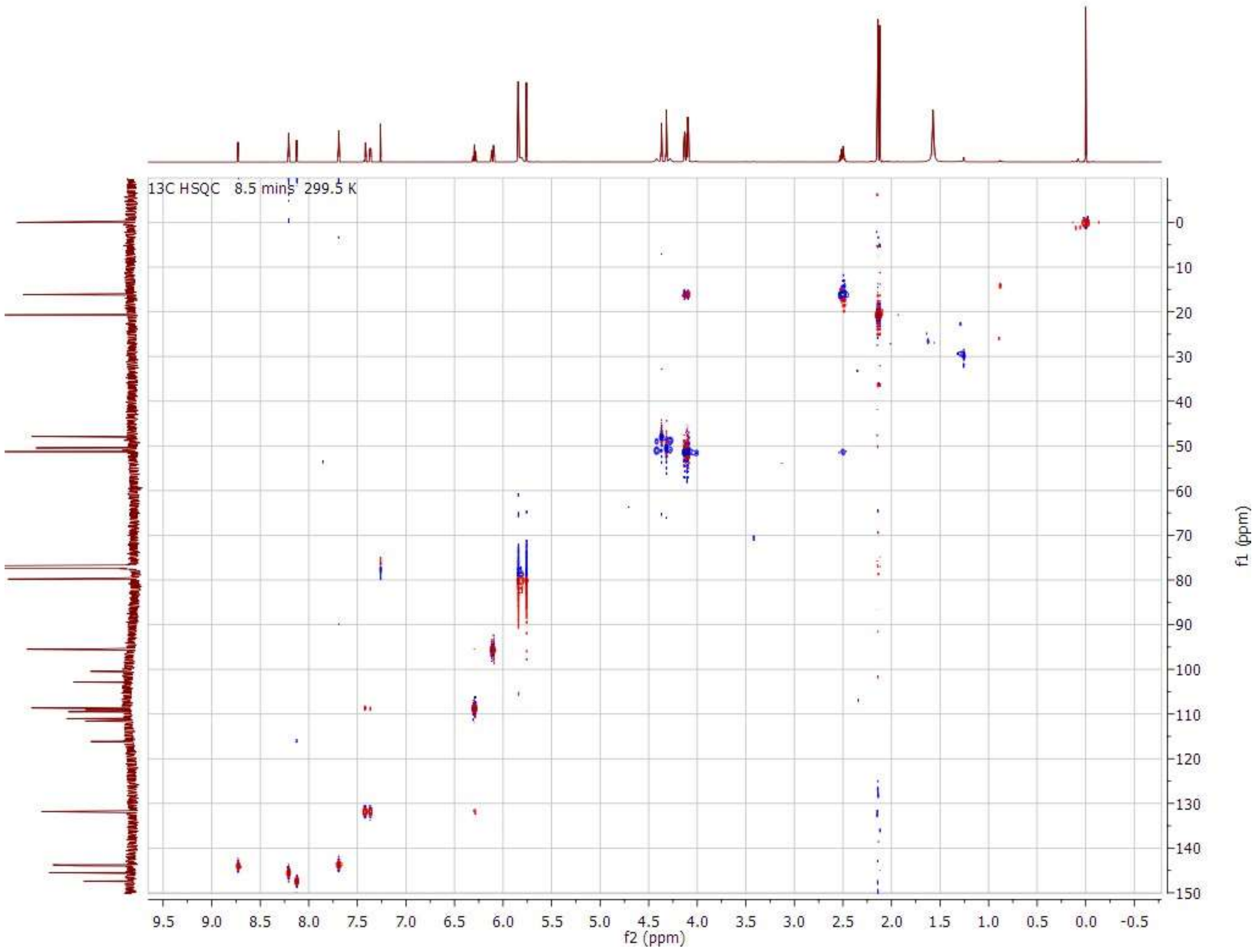

Supplementary Figure 20: ^{13}C-HSQC Spectrum of RT-AM

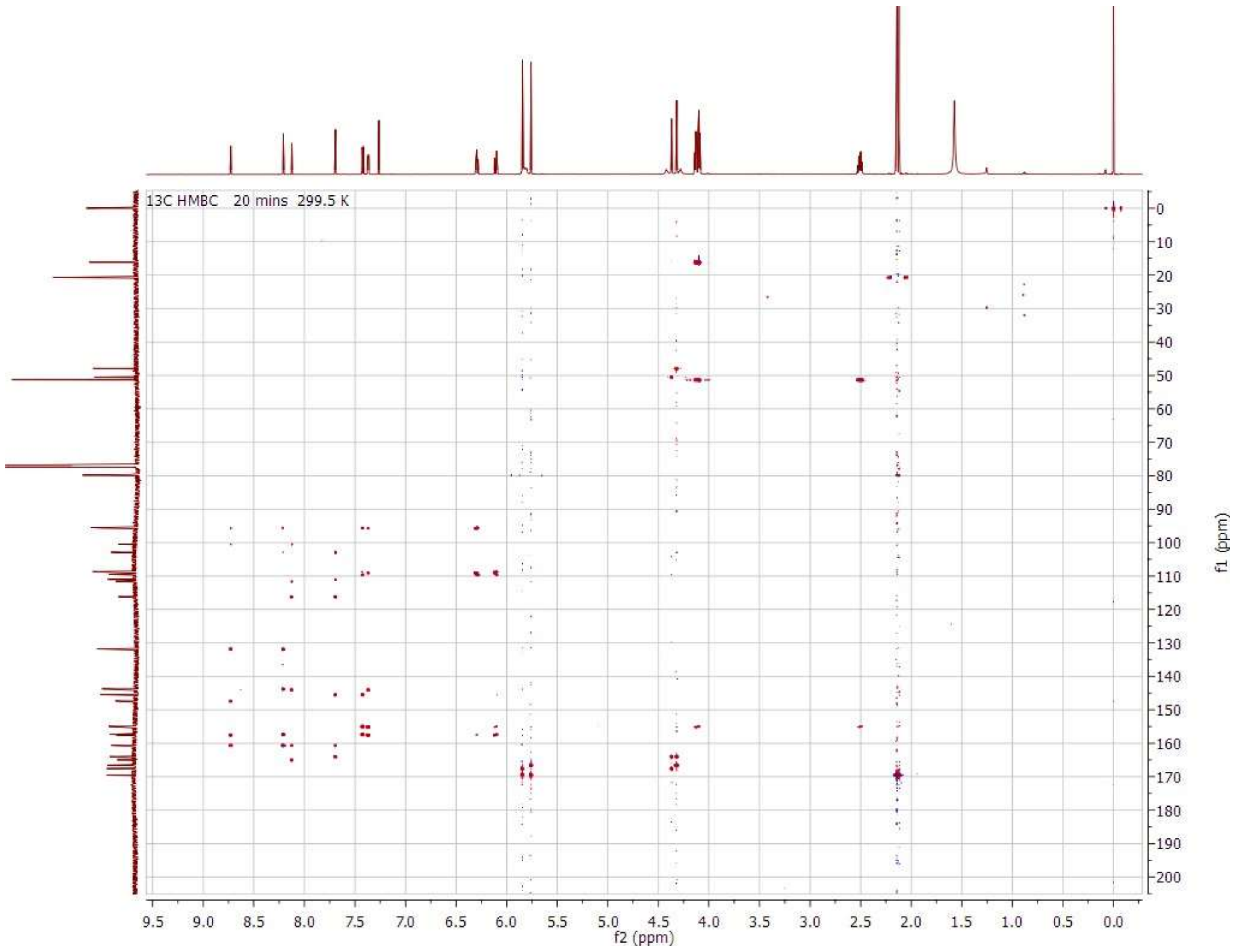

Supplementary Figure 21. ^{13}C-HMBC Spectrum of RT-AM

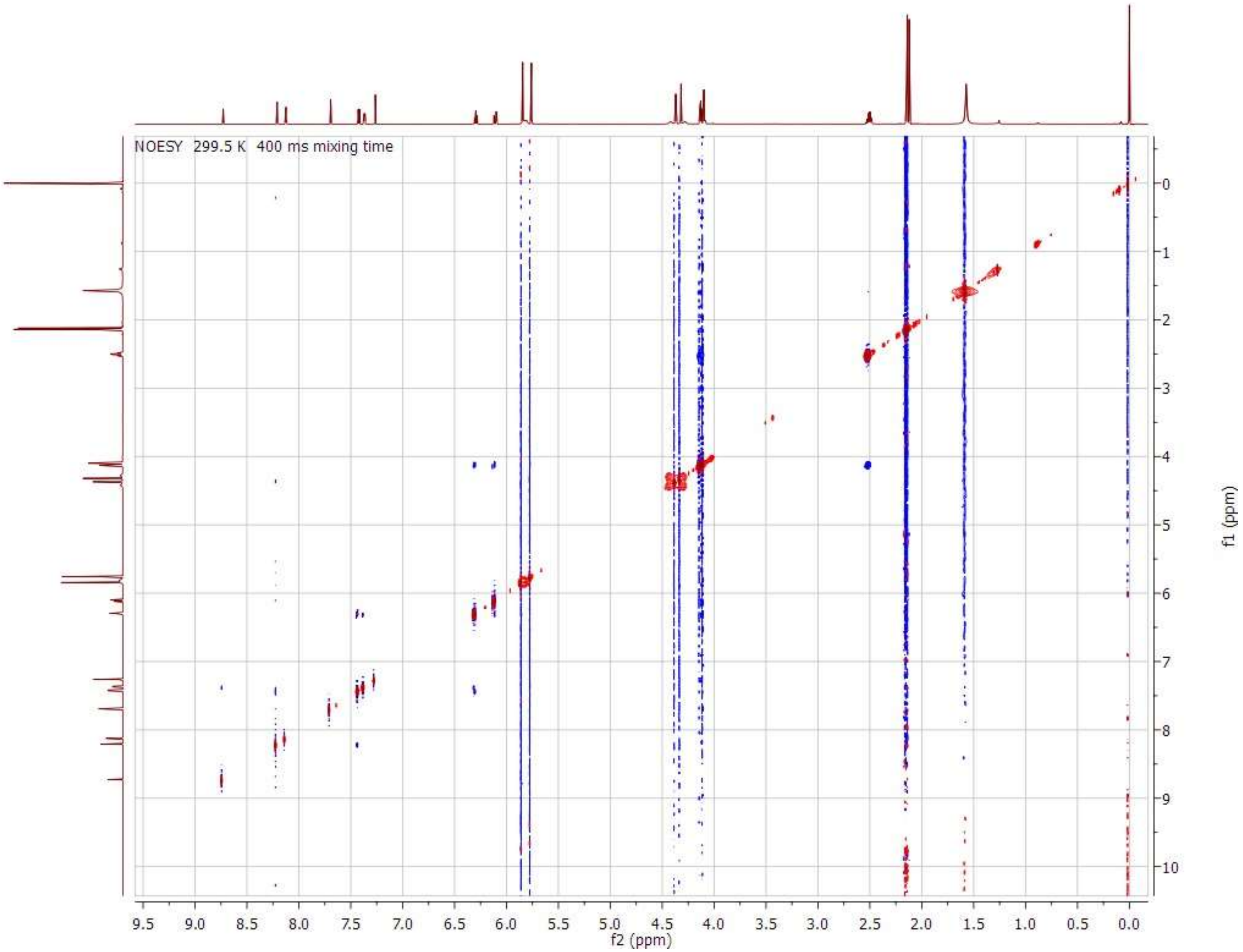

Supplementary Figure 22. NOESY Spectrum of RT-AM

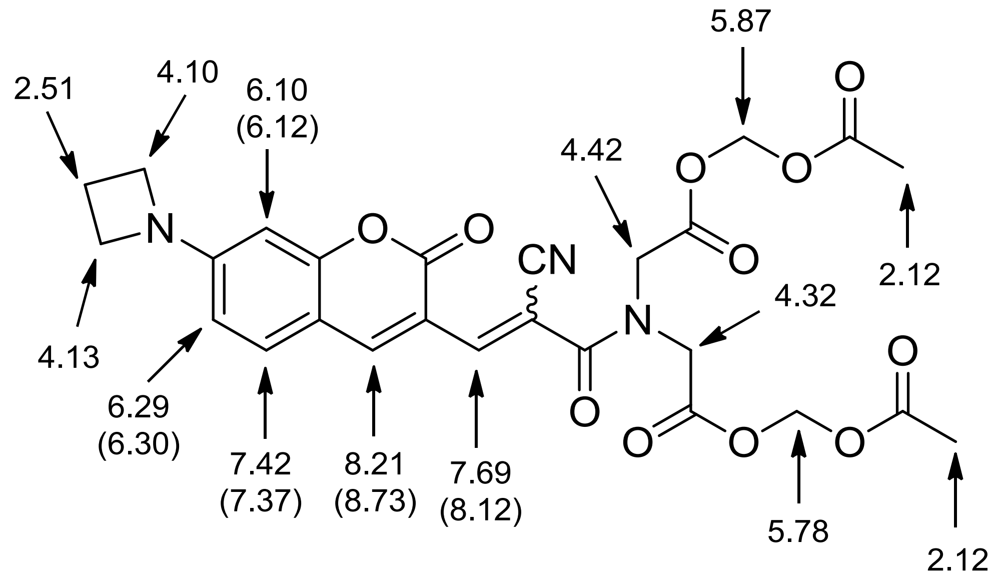

^{1}H-NMR chemical shifts assignment of RT-AM. Numbers in bracket indicate chemical shifts of minor isomer.

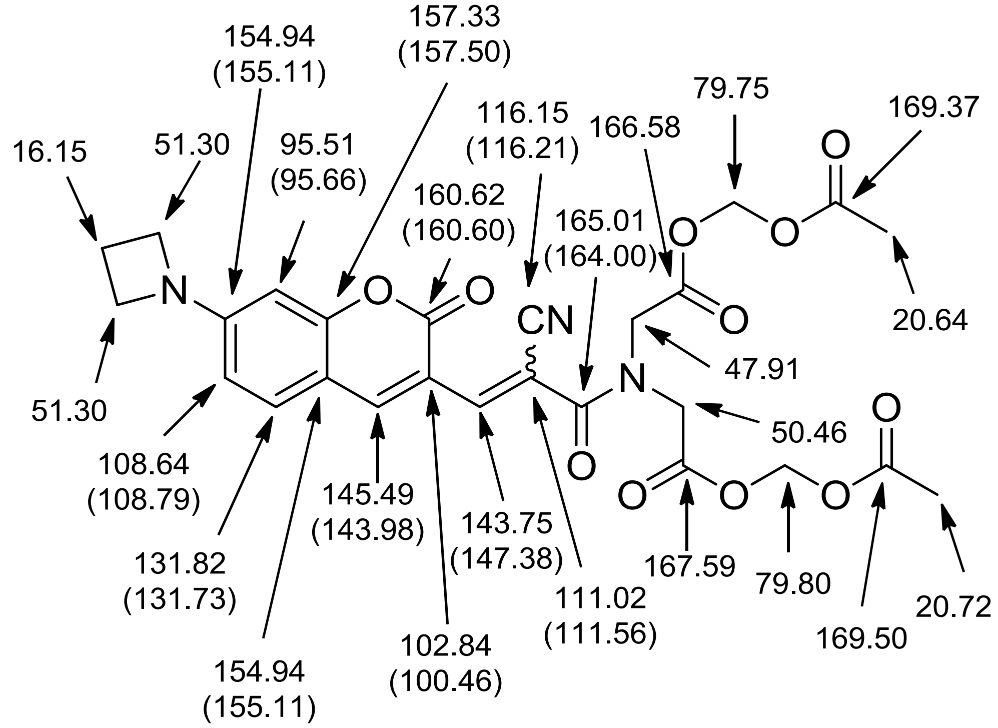

^{13}C-NMR chemical shifts assignment of RT-AM. Numbers in bracket indicate chemical shifts of minor isomer.

Supplementary Figure 23. NMR Assignments of RT-AM

Supplementary Table 1. Integration of GPC Traces

Sample	Signal Type	Peak Rage	Area*	% of Total
**Supernatant**	From Small Molecule Thiols	14.1 - 14.9 min	683	78.7
	From Protein Thiols	11.2 - 12.2 min	82.8	9.6
**Re-dissolved Pellet**	From Small Molecule Thiols	14.0 - 14.9 min	97.2	11.2
	From Protein Thiols	11.6 - 12.1 min	5.2	0.6
**Supernatant + Re-dissolved Pellet**	From Small Molecule Thiols	14.0 - 14.9 min	780	89.9
	From Protein Thiols	11.6 - 12.1 min	88.0	10.1
**Total**	All	NA	868	100.0

*Integration was calculated using the Agilent ChemStation software with manual integration

Supplementary Table 2. Image processing for GSH ratiometric images. A total of 54 areas were measured for their ratios in Supplementary Figure 4b. Results are shown in the table below. The average ratio can be then calculated from the measurements. In this specific example, the average ratio is 2.049 ± 0.307. The corresponding GSH concentration can be then calculated using a standard curve specifically generated for this experiment. Here, the calculated GSH concentration in Supplementary Figure 4b is 4.6 ± 0.7 mM. Unless otherwise specified, the method described in Supplementary Figure 4 was used for all the image analysis throughout this study.

#	Area	Mean	Min	Max	#	Area	Mean	Min	Max
1	206.802	1.818	0.394	5.406	28	164.063	1.917	0.416	5.804
2	137.868	1.789	0.626	3.075	29	152.344	1.942	0.107	7.082
3	144.762	1.842	0.283	5.588	30	220.589	2.004	0.527	6.014
4	110.295	1.662	0.355	3.476	31	176.471	2.132	0.698	8.075
5	152.344	1.908	0.485	3.914	32	164.063	1.970	0.617	6.708
6	113.741	1.471	0.292	4.991	33	144.762	2.016	0.617	6.320
7	186.122	1.940	0.262	6.142	34	227.483	2.224	0.705	9.575
8	116.499	1.774	0.233	3.413	35	248.163	2.177	0.390	5.716
9	174.403	1.815	0.000	5.070	36	152.344	2.353	0.927	13.022
10	144.762	2.337	0.894	7.223	37	176.471	1.875	0.540	4.959
11	235.755	1.950	0.076	8.101	38	165.442	2.088	0.544	5.815
12	125.460	2.279	0.624	6.814	39	269.532	2.046	0.251	9.123
13	173.025	2.199	0.647	21.294	40	179.229	2.351	0.787	8.450
14	175.782	1.544	0.366	4.316	41	165.442	2.736	1.120	7.858
15	221.279	2.286	0.000	7.277	42	232.997	1.984	0.550	5.492
16	221.968	1.850	0.396	5.734	43	227.483	1.847	0.007	5.823
17	125.460	2.285	0.834	6.138	44	198.530	1.926	0.578	7.414
18	116.499	1.792	0.614	6.375	45	157.170	2.356	0.353	6.310
19	82.721	2.515	0.659	7.911	46	154.412	2.020	0.601	4.339
20	121.324	1.754	0.364	11.733	47	73.760	3.356	0.719	48.167
21	124.081	2.198	0.762	5.633	48	111.673	1.901	0.621	5.172
22	198.530	1.970	0.483	5.937	49	106.159	1.762	0.722	3.600
23	224.036	2.050	0.631	6.599	50	116.499	1.529	0.224	3.798
24	166.821	1.974	0.336	3.668	51	175.782	1.963	0.345	6.598
25	206.802	2.045	0.758	4.984	52	103.401	2.105	0.728	6.419
26	269.532	2.219	0.598	24.800	53	165.442	2.202	0.366	4.442
27	199.220	2.305	0.793	12.000	54	165.442	2.309	0.589	5.553

Supplementary Table 3. Material Used in Deriving Neurons from Human Embryonic Stem Cells.

Neurobasal media	Invitrogen, 21103-049
DMEM/F-12 media	Invitrogen, 11330-032
B-27 Supplement	Invitrogen, 12587-010
N-2 Supplement	Invitrogen, 17502-048
Poly-L-ornithine	Sigma, P4957
Laminin	Invitrogen, 23017
bFGF	PeproTech, AF-100-18B
EGF	PeproTech, AF-100-15
BDNF	PeproTech, AF-450-02
GDNF	PeproTech, AF-450-10
NT-3	PeproTech, AF-450-03
Y-27632	Selleckchem, S1049
SB-431542	Selleckchem, S1067
Cyclopamine	Selleckchem, S1146
Dorsomorphin	Santa Cruz, sc-361173
Dibutyryl-cAMP	Santa Cruz, sc-201567
L-ascorbic acid-2-phosphate	Sigma, A8960
Accutase	Stem Cell, 07920
Rosette Selection Reagent	Stem Cell, 05832
Penicillin Streptomycin	Invitrogen, 15140-122
Matrigel, hESC-Qualified	Corning, 356277

Supplementary Table 4. Media Used in Deriving Neurons from Human Embryonic Stem Cells

Essential 8 Medium (E8)	DMEM/F12 with glutamine and HEPES, 20 µg/ml Insulin, 64 µg/ml L-ascorbic acid-2-phosphate, 14 ng/ml Sodium selenite, 10.7 µg/ml Transferrin, 100 ng/ml Human–FGF2, 2 ng/ml Human-TGF-β1, and 1% Pen/Strep
Neural Induction Medium (NIM)	Prepared from equal volumes of DMEM/F-12 and Neurobasal medium with 2% B27-supplement, 1% N2-supplement, 2 mM Glutamax, and 1% Pen/Strep
Neural Proliferation Medium (NPM)	Prepared from equal volumes of DMEM/F-12 and Neurobasal media with 1% B27-supplement, 0.5% N2-supplement, 20 ng bFGF per ml, and 20 ng EGF per ml 2 mM Glutamax, and 1% Pen/Strep
Neural Differentiation Medium (NDM)	Prepared from neurobasal medium with 2% B27, 20 ng per ml BDNF, 10 ng per ml NT-3, 10 ng per ml GDNF, 200 µM ascorbic acid and 100 µM db-cAMP, 2 mM Glutamax, and 1% Pen/Strep

Supplementary Table 5. Primers used in the Real-time PCR experiment demonstrating a high fold change in specific NPCs markers.

	Gene	Primer Sequence	Exon	Amplicon size
NPC genes	hFABP7.FOR	GCACATTCAAGAACACGGAGA	2	203
	hFABP7.REV	CACATCACCAAAAGTAAGGGTCA	4	203
	hSOX2.FOR	TACAGCATGTCCTACTCGCAG	1	110
	hSOX2.REV	GAGGAAGAGGTAACCACAGGG	1	110
	hPAX6.FOR	AACGATAACATACCAAGCGTGT	7&8	120
	hPAX6.REV	GGTCTGCCCGTTCAACATC	8	120
	hNESTIN.FOR	GGCGCACCTCAAGATGTCC	2	127
	hNESTIN.REV	CTTGGGGTCCTGAAAGCTG	3&4	127
	hDLX2.FOR	CCTACACCTCCTACGCTCC	1	93
	hDLX2.REV	TCACTATCCGAATTTCAGGCTCA	2	93
	hASCL1.FOR	GGAGCTTCTCGACTTCACCA	1	126
	hASCL1.REV	CAACGCCACTGACAAGAAAG	2	126
HK genes	hTBP.FOR	AACAACAGCCTGCCACCTTA	2	96
	hTBP.REV	GCCATAAGGCATCATTGGAC	3	96
	hGUSB.FOR	AAACGATTGCAGGGTTTCAC	10	171
	hGUSB.REV	CTCTCGTCGGTGACTGTTCA	11&12	171
IPCS gene	hNANOG.FOR	TGCAAGAACTCTCCAACATCC	2	177
	hNANOG.REV	CCTGGTGGTAGGAAGAGTAAAG	4	177

Supplementary Note 1: Deduction of Ratiometric Quantification

The reversible reaction equation is:

$$Probe(free) + Analyte = Probe(reacted)$$

or $P + A = AP$

A general equation for the relationship between ratio (R) and concentration is:

$$[A] = K_d{}'' \frac{R - R_{min}}{R_{max} - R}$$

The above equation takes into consideration spectra overlap between species P and AP.

If no spectra overlap between species P and AP, the equation should be:

$$[A] = K_d \times \frac{[AP]}{[P]}$$
$$= K_d \times R$$

In some cases, the spectra overlap is unidirectional: spectrum of AP overlaps with P, but spectrum of P does not overlap with AP, or vice versa; then either $R_{min} \to 0$ or $R_{max} \to \infty$, the equation can be simplified as:

$$[A] = K_d{}' \times R$$

It should be noted that the above equation is only valid in a certain range (linear range when $R \ll R_{max}$), and that $K_d{}'$ does not have a strict physical meaning. However, the value of $K_d{}'$ is usually in the same order of magnitude as K_d.

Additional details of the equation deduction can be found in Jiang et al. *ACS chemical biology* **10**, 864-874 (2015).

Supplementary Note 2: Influence of Instrument on Fluorescence Measurement and Ratiometric Quantitation

Different ratios can be obtained by manipulating the energy of the excitation laser and the PMT settings at the receiving end. To perform a reliable quantitation, all the calibration and measurements should be done on the same day with the same instrument and the same settings throughout the experiment. Details can be found in Jiang et al. *ACS chemical biology* **10**, 864-874 (2015).

Therefore, although the same RT solution was used for all experiments, and K_d (3.7 mM) was the same for a given chemical reaction, a different $K_d{}'$ was obtained when measuring with different instruments. We obtained $K_d{}'$=6.96 mM with the fluorometer, $K_d{}'$=3.91 mM with the plate reader, and $K_d{}'$=1~4 mM with the confocal microscope depending on the settings.

Supplementary Note 3: Chemical Synthesis and Characterization

Synthesis of compound 2

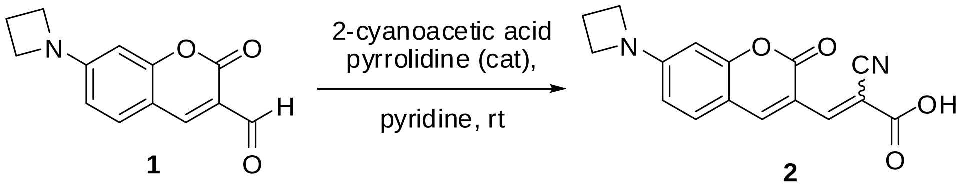

To a solution of compound **1** (22 mg, 0.096 mmol) in pyridine was added 2-cyanoacetic acid (9.8 mg, 0.115 mmol) and pyrrolidine (1.6 µL, 0.019 mmol) at room temperature.[36] The reaction was stirred at room temperature overnight. The pyridine was then evaporated under reduced pressure. Methanol was added to the residue and the mixture was filtered to afford compound **2** as a red solid (24 mg, 84%): ^{1}H NMR (400 MHz, DMSO-d_6) δ 8.74 (s, 1H), 8.22 (s, 1H), 7.59 (d, J = 8.8 Hz, 1H), 6.44 (dd, J = 8.8, 2.0 Hz, 1H), 6.27 (d, J = 1.6 Hz, 1H), 4.10 (t, J = 7.6 Hz, 4H), 2.44 – 2.35 (m, 6H), refer to Supplementary Fig. 8 for the ^{1}H NMR spectrum; ^{13}C NMR (100 MHz, DMSO-d_6) δ 164.05, 160.51, 157.57, 155.66, 147.38, 144.81, 132.68, 117.05, 110.16, 109.72, 108.91, 95.59, 51.79, 16.00, refer to Supplementary Fig. 9 for the ^{13}C NMR spectrum; HRMS (m/z): $[M+1]^+$ calcd. for $C_{16}H_{12}N_2O_4$, 297.0875, found, 297.0875; refer to Supplementary Fig. 10. for the HRMS spectrum.

Synthesis of compound 3

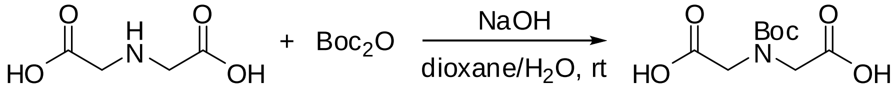

3

To a solution of iminodiacetic acid (2 g, 15 mmol) in dioxane/H_2O (3:1, 40 mL) were added Boc_2O (3.93 g, 18 mmol) and 10 mL of 2 M NaOH solution at 0 °C.[37] The mixture was stirred overnight at room temperature. The dioxane was evaporated under reduced pressure. The remaining aqueous layer was acidified with a 1 M HCl solution and extracted with AcOEt. The organic layer was dried over $MgSO_4$ and evaporated to give **3** (67%), as a white solid directly used in the next step.

Synthesis of compound 5

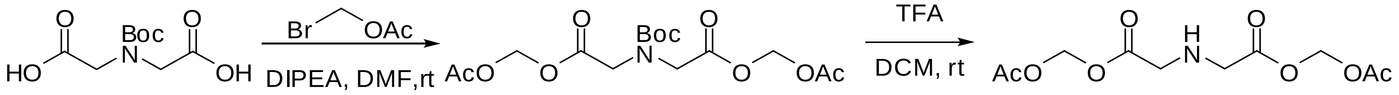

3 **4** **5**

To a solution of compound **3** (233 mg, 1.0 mmol) in anhydrous DMF (3 mL) under nitrogen protection were added bromomethyl acetate (0.392 mL, 4.0 mmol) and DIPEA (0.548 mL, 4.0 mmol). The reaction mixture was allowed to stir at room temperature overnight, and then concentrated under reduced pressure. The residue was purified by flash chromatography using (hexanes/ethyl acetate = 10:1 to 3:1) as an eluent to afford compound **4** (190 mg, 50%). ^{1}H NMR (400 MHz, $CDCl_3$) δ 5.77 (s, 2H), 5.76 (s, 2H), 4.15 (s, 2H), 4.04 (s, 2H), 2.10 (s, 6H), 1.43 (s, 9H) , refer to Supplementary Fig. 11 for the ^{1}H NMR spectrum; ^{13}C NMR (100 MHz, $CDCl_3$) δ 169.51, 169.40, 168.69, 168.49, 154.79, 81.70, 79.55, 79.43, 49.41, 48.83, 28.08, 20.69, 20.65, 20.45, refer to Supplementary Fig. 12 for the ^{13}C NMR spectrum; HRMS (m/z): $[M+1]^+$ calcd. for $C_{15}H_{23}NO_{10}$, 378.1400; found, 378.1396; refer to Supplementary Fig. 13 for the HRMS spectrum.

To the solution of compound **4** (190 mg, 0.5 mmol) in DCM (1 mL) was added TFA (1 mL). The reaction solution was stirred at room temperature for 2 h. The solvent and TFA were then removed under reduced pressure. The residual solid was diluted with saturated sodium bicarbonate solution and extracted with ethyl acetate. The combined organic layers were washed with brine, dried over anhydrous sodium sulfate, filtrated and concentrated under reduced pressure. The residue was purified by flash chromatography using (hexanes/ethyl acetate = 10:1 to 1:1) as the eluent to afford compound **5** (120 mg, 86%). ^{1}H NMR (400 MHz, $CDCl_3$) δ 5.75 (s, 4H), 3.52 (s, 4H), 2.09 (s, 6H) , refer to Supplementary Fig. 14 for the ^{1}H NMR spectrum; ^{13}C NMR (100 MHz, CDCl3) δ 170.66, 169.51, 79.30, 49.64, 20.69, refer to Supplementary Fig. 15 for the ^{13}C NMR spectrum; HRMS (m/z): $[M+1]^+$ calcd. for $C_{10}H_{15}NO_8$, 278.0876, found, 278.0883; refer to Supplementary Fig. 16 for the HRMS spectrum.

Synthesis of RT-AM

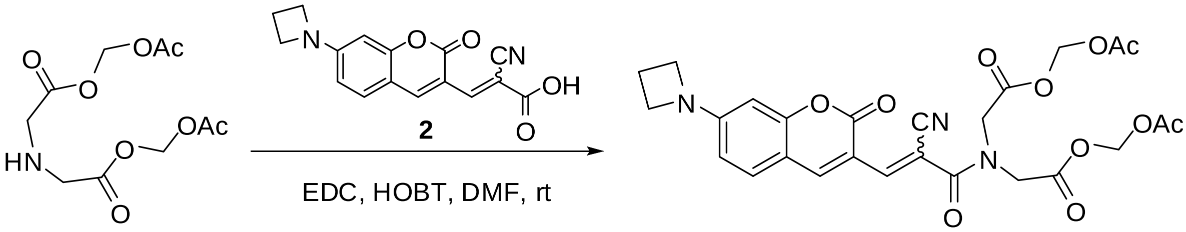

5 **RT-AM**

To a solution of compound **2** (5 mg, 0.0169 mmol) in DMF (1 mL) were added EDC (12.6 mg, 0.066 mmol), HOBt (8.9 mg, 0.066 mmol), TEA (9.2 uL, 0.066 mmol) and compound **5** (18 mg, 0.066 mmol) at room temperature. The reaction solution was stirred overnight. The solvent was removed under reduced pressure. The residual was diluted with water and extracted with ethyl acetate. The combined organic layers were washed with brine, dried over anhydrous sodium sulfate, filtrated and concentrated under reduced pressure. The residue was purified by flash chromatography using (hexanes/ethyl acetate = 10:1 to 3:1) as an eluent to afford **RT-AM** (6 mg, 64%). HPLC was used for further purification. ^{1}H NMR (800 MHz, $CDCl_3$): Minor isomer: δ 8.73 (s, 1H), 8.12 (d, J = 0.6 Hz, 1H), 7.37 (d, J = 8.8 Hz, 1H), 6.30 (dd, J = 2.2, 8.8 Hz, 1H), 6.12 (d, J = 2.2 Hz, 1H), 5.86 (s, 2H), 5.85 (s, 2H), 4.42 (s, 2H), 4.32 (s, 2H), 4.13 (t, J = 7.5 Hz, 2H), 4.10 (t, J = 7.5 Hz, 2H), 2.51 (tt, J = 7.5, 15.0 Hz, 2H), 2.14 (s, 6H). Major isomer: δ 8.21 (s, 1H), 7.69 (d, J = 0.6 Hz, 1H), 7.42 (d, J = 8.8 Hz, 1H), 6.29 (dd, J = 2.2, 8.8 Hz, 1H), 6.10 (d, J = 2.2 Hz, 1H), 5.81(s, 2H), 5.78 (s, 2H), 4.37 (s, 2H), 4.28 (s, 2H), 4.13 (t, J = 7.5 Hz, 2H), 4.10 (t, J = 7.5 Hz, 2H), 2.51 (tt, J = 7.4, 15.0 Hz, 2H), 2.12 (s, 6H). The final compound was a mixture with 56% major isomer and 44% minor isomer, refer to Supplementary Fig. 17 for the ^{1}H NMR spectrum. ^{13}C NMR (200 MHz, $CDCl_3$): Minor isomer: δ 169.50, 169.37, 167.59, 166.58, 164.00, 160.60, 157.50, 155.11, 147.38, 143.98, 131.73, 116.21, 111.56, 109.00, 108.79, 100.46, 95.66, 79.80, 79.75, 51.31 (2C), 50.46, 47.91, 20.72, 20.64, 16.12. Major isomer: δ 169.50, 169.37, 167.59, 166.58, 165.01, 160.62, 157.33, 154.94, 145.49, 143.75, 131.82, 116.15, 111.02, 109.50, 108.64, 102.84, 95.51, 79.80, 79.75, 51.31 (2C), 50.46, 47.91, 20.72, 20.64, 16.15, refer to Supplementary

Fig. 18 for the ^{13}C NMR spectrum. HRMS (m/z): $[M+1]^+$ calcd. for $C_{26}H_{25}N_3O_{11}$, 556.1567; found, 556.1564; refer to Supplementary Fig. 19 for HRMS spectrum. For 2D NMR and corresponding peak assignments, refer to Supplementary Figs. 20-23.
